# Edem1 activity in the fat body regulates insulin signalling and metabolic homeostasis in *Drosophila*

**DOI:** 10.26508/lsa.202101079

**Published:** 2021-06-17

**Authors:** Himani Pathak, Jishy Varghese

**Affiliations:** School of Biology, Indian Institute of Science Education and Research (IISER TVM) Thiruvananthapuram, Kerala, India

## Abstract

In *Drosophila*, nutrient status is sensed by the fat body, a functional homolog of mammalian liver and white adipocytes. The fat body conveys nutrient information to insulin-producing cells through humoral factors which regulate *Drosophila* insulin-like peptide levels and insulin signalling. Insulin signalling has pleiotropic functions, which include the management of growth and metabolic pathways. Here, we report that Edem1 (endoplasmic reticulum degradation–enhancing α-mannosidase–like protein 1), an endoplasmic reticulum–resident protein involved in protein quality control, acts in the fat body to regulate insulin signalling and thereby the metabolic status in *Drosophila*. Edem1 limits the fat body–derived *Drosophila* tumor necrosis factor-α Eiger activity on insulin-producing cells and maintains systemic insulin signalling in fed conditions. During food deprivation, *edem1* gene expression levels drop, which aids in the reduction of systemic insulin signalling crucial for survival. Overall, we demonstrate that Edem1 plays a vital role in helping the organism to endure a fluctuating nutrient environment by managing insulin signalling and metabolic homeostasis.

## Abstract (precis)

Edem1, an endoplasmic reticulum-resident protein, acts in the fat body to regulate insulin signalling and metabolic status in Drosophila, which aids in survival against nutrient deprivation.

## Introduction

Energy homeostasis, the sum of all processes which maintain the balance between energy inflow and outflow; is vital for normal functioning, reproduction as well as longevity. Energy homeostasis in animals is brought about by the activity and interplay of various endocrine and neuroendocrine systems. Insulin/insulin-like growth factor (IGF) signalling pathway plays a significant role in the maintenance of energy balance and is well conserved in both vertebrates and invertebrates (Kenyon et al, 1993; Kimura et al, 1997; Brogiolo et al, 2001; Clancy et al, 2001; Fabrizio et al, 2001; Britton et al, 2002; Fernandez & Torres-Aleman, 2012). The perturbations in insulin signalling result in a plethora of effects, such as; diabetes (Britton et al, 2002; Rulifson et al, 2002), obesity (Kahn et al, 2006), reduced body size (Liu et al, 1993; Accili et al, 1996; Ikeya et al, 2002; Rulifson et al, 2002), resistance to starvation and oxidative stress, and life span effects (Clancy et al, 2001; Tatar et al, 2001; Bonafè et al, 2003; Holzenberger et al, 2003; Shimokawa et al, 2003; Giannakou et al, 2004; Katic & Kahn, 2005; Sonntag et al, 2005; Giannakou & Partridge, 2007). *Drosophila melanogaster*, a widely used genetic model organism, has eight insulin-like peptides (*Drosophila* insulin-like peptide [DILPs] 1–8), which share structural and functional similarities with mammalian insulin and IGFs (Grönke et al, 2010). Among these DILPs; DILP2, DILP3, and DILP5 are produced mainly by a subset of the median neurosecretory cells (mNSCs), the insulin-producing cells (IPCs), in the fly brain (Ikeya et al, 2002; Géminard et al, 2009; Broughton et al, 2010; Nässel, 2012). The major effector tissue of insulin signalling is the fat body, which is also the main energy reserve and nutrient sensor in flies (Hwangbo et al, 2005; Géminard et al, 2009). The fat body relays information about the nutrient status of the organism through humoral factors, which act on the IPCs directly or indirectly to control systemic insulin signalling (Colombani et al, 2003; Géminard et al, 2009; Droujinine & Perrimon, 2016). The fat-derived signals that control IPC function include DILP6 (Bai et al, 2012), a DILP; unpaired2 (Upd2), a functional homolog of leptin in *Drosophila* and activator of JAK-STAT pathway (Rajan & Perrimon, 2013); Eiger, the *Drosophila* tumor Necrosis Factor α/TNFα, which activates JNK signalling (Agrawal et al, 2016); CCHamide2 (Sano et al, 2015), a nutrient responsive peptide hormone; growth-blocking peptide (GBP) (Koyama & Mirth, 2016), a *Drosophila* cytokine; Stunted, a circulating insulinotropic peptide (Delanoue et al, 2016); female-specific independent of transformer (FIT) (Sun et al, 2017); and activin-like ligand Dawdle (Ghosh & O’Connor, 2014). The molecular mechanisms that regulate the synthesis and secretion of the fat body–derived signals (FDSs) are currently under intense investigation.

The ER serves many functions in the eukaryotic cell, foremost of which is the synthesis and folding of nascent proteins with the help of molecular chaperones and folding enzymes. Hence, the ER is considered as the major quality-control site which ensures that only correctly folded proteins are allowed to leave to other cellular compartments. The ER is also considered to be the first storage site of secretory proteins and the ER activity is high in cells of endocrine and exocrine tissues because of the heavy protein trafficking in such cells. Genetic factors, physiological changes, and fluctuations in the cellular environment might lead to misfolding of proteins (Liu & Kaufman, 2003) and the ER aids in eliminating proteins, which remain misfolded even after multiple rounds of folding attempts. Thus, a proper balance between the influx of proteins and the folding machinery in the ER is crucial for efficient protein quality control. When the ER homeostasis is upset misfolded proteins accumulate in the ER triggering an adaptive response called unfolded protein responses (UPR). The UPR signalling mainly involves three ER residing transmembrane sensors: inositol-requiring protein 1, activating transcription factor 6, and PKR-like ER kinase (PERK). The UPR sensors would initiate ER-associated degradation (ERAD) of terminally misfolded proteins, expand the ER membrane, increase the folding capacity of the ER, and decrease the overall protein load in the ER (Liu & Kaufman, 2003). Permanently unfolded glycoproteins are recognised by ERAD-enhancing α-mannosidase–like proteins (Edem), which aid in the degradation of the misfolded proteins (Molinari et al, 2003; Araki & Nagata, 2011; Kroeger et al, 2012). Glycoproteins constitute a large proportion of proteins in a cell; hence, the function of Edem is crucial for cellular homeostasis.

Here, we report that Edem1 activity in the *Drosophila* fat body is crucial for maintaining systemic insulin signalling. Down-regulation of *edem1* gene expression in the fat body resulted in the accumulation of DILP2 in the IPCs, a decrease in *dilp3* mRNA levels and reduced systemic insulin signalling, which led to nutrient imbalances and altered sensitivity to starvation. Our results also show that Edem1 regulates fat body–derived *Drosophila* TNFα Eiger activity on the IPCs, crucial for managing systemic insulin signalling and metabolic status. Activation of target of rapamycin (TOR) signalling, the main amino acid sensor, and a key regulator of Eiger activity rescued the effects of *edem1* down-regulation. In addition, we report that Edem1 activity in the fat body regulate Upd2, another fat body–derived cytokine, to manage metabolic status. Furthermore, in response to nutrient deprivation, *edem1* transcripts were found to be low, which we show is critical to the reduction in systemic insulin levels and better survival of flies during starvation. We propose that Edem1 acts as a key factor in the fat body, which maintains nutrient homeostasis by controlling the activity of the IPCs through Eiger.

## Results

### Edem1 maintains metabolic homeostasis

**Figure 1.**
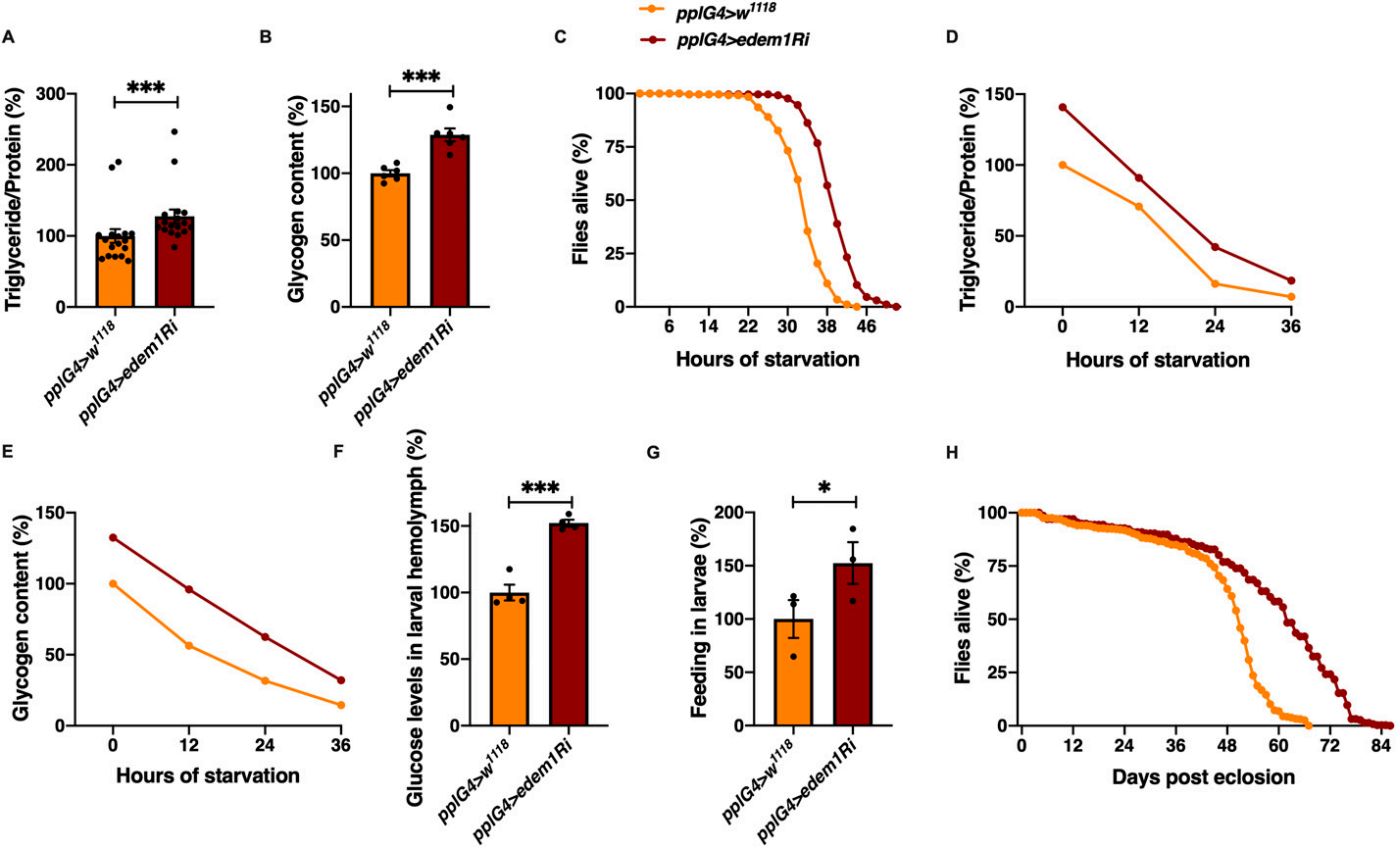
Edem1 maintains metabolic homeostasis. **(A)** Blocking *edem1* expression using RNAi in the fat body led to enhanced triglyceride levels in adult 5-d-old male flies. Data are shown as % ratio of triglyceride to total protein levels, normalised to 100% in *pplGal4>w*^*1118*^ (control) and increase in experimental conditions *pplGal4>UAS-edem1-RNAi* (independent biological replicates = 17, *P*-value between control and *UAS-edem1-RNAi* is <0.001 [Mann–Whitney test]). **(B)** Enhanced levels of glycogen in adult 5-d-old male flies caused by blocking *edem1* expression in the fat body. Data are shown as % of total glycogen levels, normalised to 100% in *pplGal4>w*^*1118*^ (control), and increase in experimental conditions *pplGal4>UAS-edem1-RNAi* (independent biological replicates = 6, *P*-value between control and *UAS-edem1-RNAi* is <0.001 [Mann–Whitney test]). **(C)** Enhanced resistance to starvation in adult 5-d-old male flies caused by blocking *edem1* expression in the fat body. Data shown as percentage of flies of *pplGal4>w*^*1118*^ (control) and *pplGal4>UAS-edem1-RNAi* which were alive at various time points of starvation (independent biological replicates = 4, number of flies used for control is 255 and for *pplGal4>UAS-edem1-RNAi* is 262. *P*-value between control and *UAS-edem1-RNAi* is <0.001 [log-rank test], Wald test = 189.8 on df = 1, *P* < 0.001 [Cox hazard proportional analysis]). **(D, E)** Utilisation of triglycerides and glycogen at different stages of starvation upon *edem1* knock down. Data are shown as % ratio of triglyceride to total protein levels in adult male flies, data are normalised to 100% in *pplGal4>w*^*1118*^ (control) fed condition and change in response to indicated hours of starvation in control and experimental conditions *pplGal4>UAS-edem1-RNAi* is shown (independent biological replicates = 3, *P*-value between control and *UAS-edem1-RNAi* is 0.3844 [log-rank test]). Glycogen levels at different stages of starvation upon *edem1* knock down. Data are normalised to 100% in *pplGal4>w*^*1118*^ (control) fed condition and change in response to indicated hours of starvation in control and experimental conditions *pplGal4>UAS-edem1-RNAi* is shown (independent biological replicates = 3, *P*-value between control and *UAS-edem1-RNAi* is 0.0082 [log-rank test]). **(F)** Expression of *edem1*-RNAi in the fat body led to enhanced glucose levels in the circulation. Data are shown as % of glucose levels in the hemolymph, normalised to 100% in *pplGal4>w*^*1118*^ (control) and increase in experimental conditions *pplGal4>UAS-edem1-RNAi* (independent biological replicates = 4, *P*-value between control and *UAS-edem1-RNAi* is <0.001 [Mann–Whitney test]). **(G)** Blocking *edem1* gene expression in the fat body led to enhanced feeding responses in larvae. Data are shown as % food consumption in larvae, normalised to 100% in *pplGal4>w*^*1118*^ (control) and increase in experimental conditions *pplGal4>UAS-edem1-RNAi* (independent biological replicates = 3, *P*-value between control and *UAS-edem1-RNAi* is 0.0329 [Welch’s *t* test]). **(H)** *edem1*-RNAi in the fat body led to enhanced life span in adult male flies. Data are shown as percentage of input flies *pplGal4>w*^*1118*^, *pplGal4>UAS-edem1-RNAi* which were alive across the days (independent biological replicates = 3, number of flies used for control is 453 and for *pplGal4>UAS-edem1-RNAi* is 372. *P*-value between control and *UAS-edem1-RNAi* is <0.001 [log-rank test], Wald test = 275.1 on df = 1, *P* < 0.001 [Cox hazard proportional analysis]). **(A, B, F, G)** (*P*-value *<0.05; ** <0.01, *** <0.001; Data information: In [A, B, F, G] data are presented as mean ± SEM). Source data are available for this figure.

**Figure S1.**
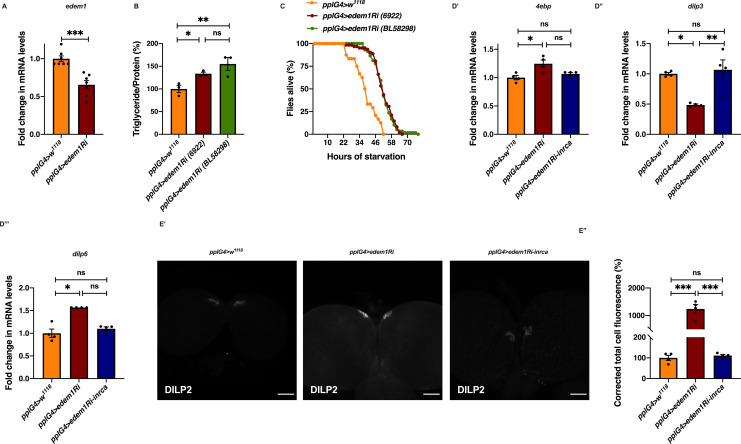
Fold change in edem1 levels in response to edem1-RNAi expression in the fat body. **(A)** Down-regulation of *edem1* in the fat body led to decreased levels of *edem1* mRNA when compared to control. Data are normalised to *pplGal4>w*^*1118*^ and fold change in *pplGal4>UAS-edem1-RNAi* is shown (independent biological replicates = 7 *P*-value between control and *UAS-edem1-RNAi* is 0.0006 [Mann–Whitney test]). **(B)** Blocking *edem1* expression using *UAS-edem1-RNAi* (VDRC_6922) and *UAS-edem1-RNAi* (RRID:BDSC_58298) in the fat body led to enhanced triglyceride levels in adult male flies. Data are shown as % ratio of triglyceride to total protein levels, normalised to 100% in *pplGal4>w*^*1118*^ (control), and increase in experimental conditions *pplGal4> UAS-edem1-RNAi* (VDRC_6922) and *UAS-edem1-RNAi* (RRID:BDSC_58298) (independent biological replicates = 3, *P*-value between control and *UAS-edem1-RNAi* [VDRC_6922] is 0.0410, *P*-value between control and *UAS-edem1-RNAi* [RRID:BDSC_58298] is 0.0013 and *P*-value between *UAS-edem1-RNAi* [VDRC_6922] and *UAS-edem1-RNAi* [RRID:BDSC_58298] is 0.3356 [Kruskal–Wallis test followed by Dunn’s post hoc test]). **(C)** Enhanced resistance to starvation in adult male flies caused by blocking *edem1* using *UAS-edem1-RNAi* (VDRC_6922) and *UAS-edem1-RNAi* (RRID:BDSC_58298) expression in the fat body. Data shown as percentage of flies of *pplGal4>w*^*1118*^ (control) and *UAS-edem1-RNAi* (VDRC_6922) and *UAS-edem1-RNAi* (RRID:BDSC_58298) which were alive at various time points of starvation (independent biological replicates = 3, number of flies used for control is 24, for *pplGal4>UAS-edem1-RNAi* [6922] is 62 and for *pplGal4>UAS-edem1-RNAi* [BL58298] is 59. *P*-value between control and *UAS-edem1-RNAi* [VDRC_6922] is <0.001, *P*-value between control and *UAS-edem1-RNAi* [RRID:BDSC_58298] is <0.001 and *P*-value between *UAS-edem1-RNAi* [VDRC_6922] and *UAS-edem1-RNAi* [RRID:BDSC_58298] is 0.8256 [log-rank test], Wald test = 14.11 on df = 1, *P* < 0.001 [Cox proportional hazard analysis]). **(D)** Increase in *4ebp* and *dilp6* and decrease in *dilp3* in response to *edem1*-RNAi was rescued by expressing *UAS-InR*^*A1325D*^ in the fat body (D). Data are shown as fold change in mRNA levels, values are normalised to *pplGal4>w*^*1118*^, and fold change in *pplGal4>UAS-edem1-RNAi* and *pplGal4> UAS-edem1-RNAi*; *UAS-InR*^*A1325D*^ is shown (independent biological replicates = 4. *P*-value between control and *UAS-edem1-RNAi* is 0.0133 for *4ebp*, 0.0108 for *dilp3*, 0.0211 for *dilp6*. *P*-value between control and *UAS-edem1-RNAi*; *UAS-InR*^*A1325D*^ is 0.5334 for *4ebp*, 0.8764 for *dilp3*, >0.9999 for *dilp6*. *P*-value between *UAS-edem1-RNAi* and *UAS-edem1-RNAi*; *UAS-InR*^*A1325D*^ is 0.4030 for *4ebp*, 0.0052 for *dilp3*, 0.1082 for *dilp6* [Kruskal–Wallis test followed by Dunn’s post hoc test for *4ebp* and *dilp6*, Ordinary one-way ANOVA followed by Tukey’s post hoc test for *dilp3*]). **(D’, E)** DILP2 levels in the insulin-producing cells in response to *edem1-RNAi* were rescued by expressing *UAS-InR*^*A1325D*^ in the fat body (D’). Shown are representative images of anti-DILP2 antibody staining in larval brains of *pplGal4>w*^*1118*^, *pplGal4>UAS-edem1-RNAi* and *pplGal4>UAS-edem1-RNAi*; *UAS-InR*^*A1325D*^. Corrected total cell fluorescence values are normalised to *pplGal4>w*^*1118*^ and fold change in *pplGal4>UAS-edem1-RNAi* and *pplGal4> UAS-edem1-RNAi*; *UAS-InR*^*A1325D*^ is shown in (D’’) (independent biological replicates = 4. *P*-value between control and *UAS-edem1-RNAi* is <0.001, *P*-value between control and *UAS-edem1-RNAi*; *UAS-InR*^*A1325D*^ is >0.9999 and *P*-value between *UAS-edem1-RNAi* and *UAS-edem1-RNAi*; *UAS-InR*^*A1325D*^ is <0.001 [Kruskal–Wallis test followed by Dunn’s post hoc test]) (Scales: 50 µm). **(A, B, D, E’’)** (*P*-value *<0.05; ** <0.01, *** <0.001; Data information: In [A, B, D, E’’], data are presented as mean ± SEM). Source data are available for this figure.

We embarked on a large-scale genetic screen in *Drosophila* to identify factors that control nutrient homeostasis and insulin signalling. Towards this, we blocked various candidate genes, reported to be differentially expressed in the *miR-14* mutants that exhibited metabolic imbalances, in the *Drosophila* fat body using RNAi lines (Varghese et al, 2010). We chose male flies for this study to minimize the effects of oogenesis on nutrient homeostasis. In this screen, we identified Edem1, an ER-resident protein involved in protein quality control, as a putative regulator of metabolic status in *Drosophila*. Down-regulation of *edem1* transcripts in the fat body led to a significant increase in the levels of energy stores—triglycerides and glycogen—in 5-d-old adult flies (Fig 1A and B). In response to knock down of *edem1* in the fat body, flies survived longer in response to acute nutrient deprivation (Fig 1C). We chose 5-d-old flies to completely avoid the influence of larval fat cells which persists in adult flies for few days after eclosion. We have confirmed the effects of blocking Edem1 in the fat body using independent RNAi lines, which rules out off-target effects and insertional site-specific effects (Fig S1B and C). We have also down-regulated *edem1* expression with an independent fat body driver *CgGal4* to make sure that the effect is coming because of fat body–specific knock down of *edem1* (Fig S4A–H). We could replicate most of the experiments from Fig 1 with the *CgGal4* driver as well. The higher energy stores present in response to reduction in *edem1* levels in the fat body, and excess energy stores mobilized could account for the better survival of flies during nutrient deprivation (Fig 1D and E). Along with changes in stored nutrient levels in adult flies, circulating glucose levels were high in the larval hemolymph (Fig 1F). In addition, blocking *edem1* in the fat body led to enhanced feeding responses in the larvae (Fig 1G), similar to responses reported earlier in food deprived larvae and also in response to low insulin because of its anorexigenic effects (Zhang et al, 2013; Chouhan et al, 2017). We also observed an increase in life span of the adult flies upon *edem1* down-regulation in the fat body (Fig 1H). These data show that Edem1 function in the fat body is crucial in regulating metabolic homeostasis in *Drosophila*. The phenotypes observed in response to blocking *edem1* levels on larval circulating sugar levels, larval feeding, adult energy stores, and life span indicated a reduction in insulin signalling, as reported by earlier studies. Reduction in the levels of DILPs produced by IPCs led to the accumulation of triglycerides and glycogen (Grönke et al, 2010; Bai et al, 2012). Ablation of IPCs also resulted in higher levels of circulating sugars, glycogen, lipid stores, and enhanced resistance to food deprivation (Rulifson et al, 2002; Broughton et al, 2005; Haselton et al, 2010). In addition, there is proof that defects in insulin signalling led to reduced adult body size and excess fat storage (Böhni et al, 1999; Tatar et al, 2001; Shingleton et al, 2005; Slaidina et al, 2009). miR-278 mutants have elevated *dilp2*, *3*, *and 5* transcript levels and are lean, whereas *miR-14* mutants are obese, because of reduced *dilp* transcript levels and insulin signalling (Teleman et al, 2006; Varghese et al, 2010). In addition, reduced insulin signalling is crucial for starvation triggered foraging and activation of IPCs or overexpression of DILPs led to less food intake (Wu et al, 2005; Hong et al, 2012). Hyperactivation of insulin signalling induced satiation in larvae (Britton et al, 2002). Also, reduced insulin signalling (Clancy et al, 2001; Tatar et al, 2001; Bai et al, 2012) and ablation of IPCs, extended life span (Broughton et al, 2005; Haselton et al, 2010). However, we did not observe any developmental growth effects as expected in response to reduced insulin signalling. Next, we tested if insulin signalling is reduced in response to blocking *edem1* levels in the fat body.

### Edem1 function in the fat body maintains systemic insulin signalling

**Figure 2.**
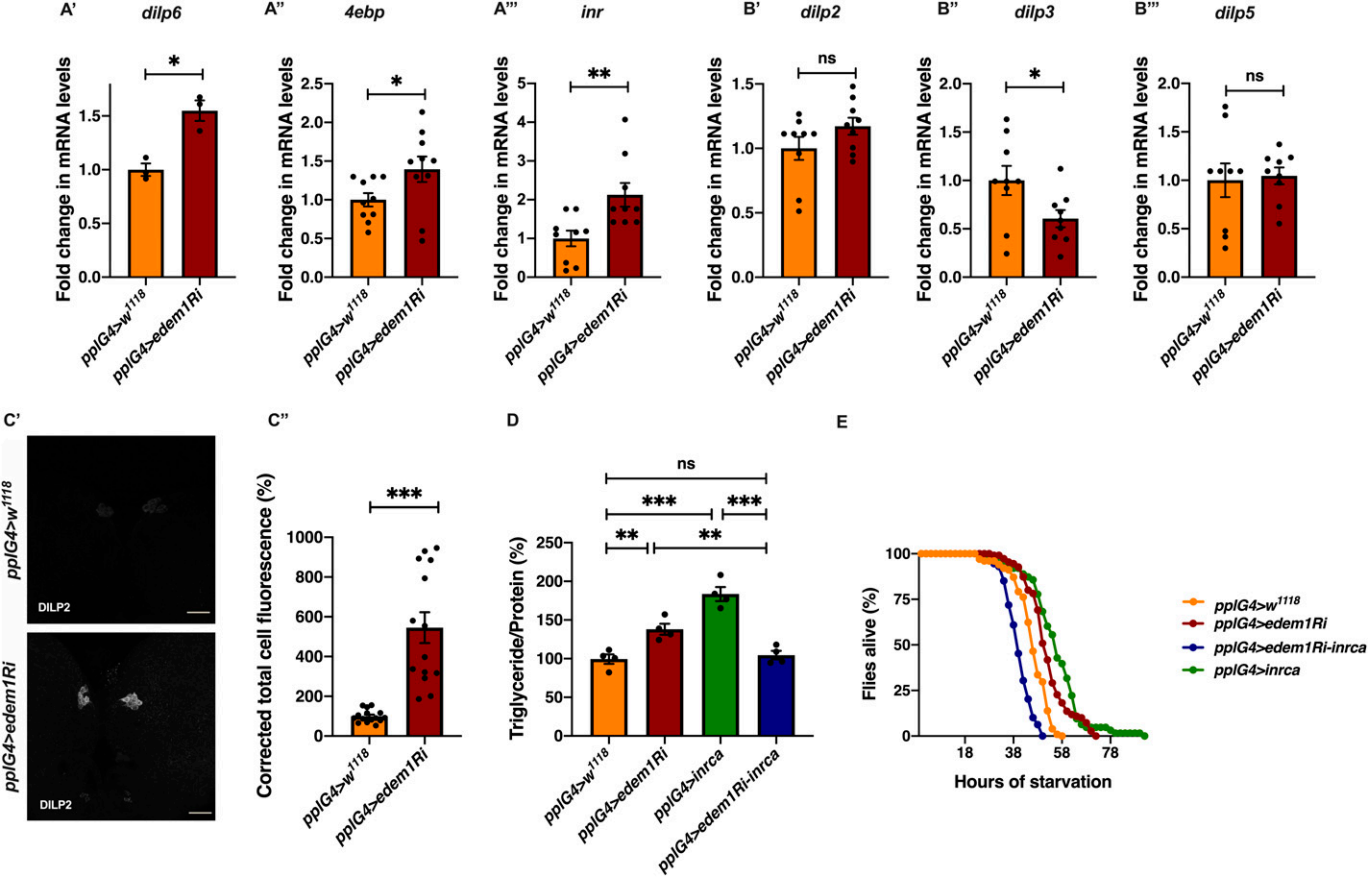
Blocking *edem1* in the fat body reduced insulin signalling. **(A)** Blocking *edem1* expression using RNAi in the fat body led to an increase in mRNA levels of insulin target genes *dilp6* (A’), *4ebp* (A’’) and *inr* (A’’’) in larvae. Data are shown as fold change in mRNA levels, values are normalised to *pplGal4>w*^*1118*^, and fold change in *pplGal4>UAS-edem1-RNAi* is shown (independent biological replicates = 3, *P*-value between control and *UAS-edem1-RNAi* is 0.0128 for *dilp6* [Welch’s *t* test], for *4ebp* independent biological replicates = 10 and *P*-value is 0.0473 [unpaired *t* test], for *inr* independent biological replicates = 9 and *P*-value is 0.0087 [Welch’s *t* test]). **(B)** Blocking *edem1* expression using RNAi in the fat body also led to a decrease in the levels of insulin-producing cell specific *dilp3* mRNA in larvae. Data are shown as fold change in mRNA levels, values are normalised to *pplGal4>w*^*1118*^, and fold change in *pplGal4>UAS-edem1-RNAi* is shown. (n = 9, *P*-value between control and *UAS-edem1-RNAi* is 0.1809 for *dilp2* [Mann–Whitney test], 0.0432 for *dilp3* [Welch’s *t* test] and 0.8187 for *dilp5* [Welch’s *t* test]). **(C)** DILP2 protein in the larval insulin-producing cells shown as a representative image (C’) of anti-DILP2 antibody staining in larval brains of *pplGal4>w*^*1118*^ (independent biological replicates = 15) and *pplGal4>UAS-edem1-RNAi* (independent biological replicates = 14). Corrected total cell fluorescence values are normalised to *pplGal4>w*^*1118*^, and fold change in *pplGal4>UAS-edem1-RNAi* is shown in (C’’) (*P*-value between control and *UAS-edem1-RNAi* is <0.001 [Mann–Whitney test]) (Scales: 30 µm). **(D)** Overexpression of a constitutively active form of *inr* (*InR*^*A1325D*^) with *edem1*-RNAi in the fat body led to the rescue of fat phenotype in adult male flies. Data is shown as % ratio of triglyceride to total protein levels, normalised to 100% in *pplGal4>w*^*1118*^ (control) and changes in experimental conditions *pplGal4>UAS-edem1-RNAi*, *pplGal4>UAS-InR*^*A1325D*^ and *pplGal4>UAS-edem1-RNAi*; *UAS-InR*^*A1325D*^ (independent biological replicates = 4, *P*-value between control and *UAS-edem1-RNAi* is <0.001, *P*-value between control and *UAS*-*InR*^*A1325D*^ is <0.001, *P*-value between *UAS-edem1-RNAi* and *UAS-edem1-RNAi*, *UAS*-*InR*^*A1325D*^ is 0.001, *P*-value between *UAS-edem1-RNAi* and *UAS*-*InR*^*A1325D*^ is 0.1292, *P*-value between *UAS*-*InR*^*A1325D*^ and *UAS-edem1-RNAi*, *UAS*-*InR*^*A1325D*^ is <0.001 and *P*-value between control and *UAS-edem1-RNAi*, *UAS*-*InR*^*A1325D*^ is >0.9999 [Kruskal–Wallis test followed by Dunn’s post hoc test]). **(E)** Starvation resistance in adult 5-d-old male flies shown as percentage of input flies *pplGal4>w*^*1118*^, *pplGal4>UAS-edem1-RNAi*, *pplGal4>UAS*-*InR*^*A1325D*^ and *pplGal4> UAS-edem1-RNAi*, *UAS*-*InR*^*A1325D*^ which were alive at various time points of starvation (independent biological replicates = 3, number of flies used for control is 77, for *pplGal4>UAS-edem1-RNAi* is 110, for *pplGal4>UAS*-*InR*^*A1325D*^ is 126 and for *pplGal4> UAS-edem1-RNAi*, *UAS*-*InR*^*A1325D*^ is 128. *P*-value between control and *UAS-edem1-RNAi* is <0.001, *P*-value between control and *UAS*-*InR*^*A1325D*^ is <0.001, *P*-value between *UAS-edem1-RNAi* and *UAS-edem1-RNAi*, *UAS*-*InR*^*A1325D*^ is <0.001, *P*-value between *UAS-edem1-RNAi* and *UAS*-*InR*^*A1325D*^ is 0.0038, *P*-value between *UAS*-*InR*^*A1325D*^ and *UAS-edem1-RNAi*, *UAS*-*InR*^*A1325D*^ is <0.001 and *P*-value between control and *UAS-edem1-RNAi*, *UAS*-*InR*^*A1325D*^ is <0.001 [log-rank test], Wald test = 10.53 on df = 1, *P* = 0.001 [Cox proportional hazard analysis]). **(A, B, C’’, D)** (*P*-value *<0.05; ** <0.01, *** <0.001; Data information: In [A, B, C’’, D] data are presented as mean ± SEM). Source data are available for this figure.

To measure the insulin signalling activity in response to blocking *edem1* in the fat body, we checked gene expression of key downstream target genes of insulin pathway. Transcription of *4ebp* (*eIF4E-binding protein*), *inr* (*insulin receptor*), and *dilp6* is suppressed by insulin signalling and these insulin target genes can be used as a read out for insulin signalling activity (Puig et al, 2003; Slaidina et al, 2009). Blocking *edem1* in the fat body increased transcript levels of the insulin responsive genes, which indicate low insulin signalling (Fig 2A). We speculated if Edem1 activity in the fat body could regulate IPC function and control systemic insulin signalling, as fat body is known to remotely control IPCs. To address whether Edem1 in the fat body regulates IPC function, the transcript levels of IPC specific DILPs—*dilp2*, *dilp3*, and *dilp5* were measured in the late third instar larval stage. In response to the expression of *edem1*-RNAi in the fat body, *dilp3* mRNA levels were found to be low; however, there were no detectable changes in the mRNA levels of *dilp2* and *dilp5* (Fig 2B). Previous studies report that nutrient deprivation would block DILP secretion from the IPCs into the hemolymph leading to an accumulation of DILPs and reduction in systemic insulin signalling (Géminard et al, 2009). We observed an increase in DILP2 puncta in IPCs in response to reducing *edem1* levels in the fat body when compared to that of control (Fig 2C’ and C’’), which suggested an accumulation of DILP2 protein in the IPCs. Together, these observations suggest that *edem1* function in the fat body maintains systemic insulin signalling in the larvae by the regulation of IPC activity.

As the next approach, we tested whether the reduction in insulin signalling in response to blocking *edem1* in the fat body was responsible for the metabolic phenotypes. A constitutively active form of insulin receptor (*InR*^*A1325D*^) was co-expressed with *edem1*-RNAi in the fat body. *InR*^*A1325D*^, which harbours an Ala–Asp mutation at position 1325, would activate downstream insulin signalling independent of DILP ligand and hence should alleviate phenotypes caused by low insulin signalling (Broughton et al, 2005; Tettweiler et al, 2005; DiAngelo & Birnbaum, 2009; Kannan & Fridell, 2013). As expected, expression of *InR*^*A1325D*^ was sufficient to alleviate high triglyceride levels and starvation resistance observed in response to knock down of *edem1* levels in the fat body (Fig 2D and E). We have performed experiments using UAS-*control* transgenes to rule out the effect of Gal4 titration in this experiment and all future rescue experiments where multiple UAS transgenes are driven by the same Gal4 driver (data not shown). These experiments show that *InR*^*A1325D*^ expression in the fat body alleviated the decrease in insulin signalling, caused by blocking *edem1*, as shown by *4ebp* and *dilp6* mRNA levels (Fig S1D’ and D’’’). Reduced *dilp3* levels (Fig S1D’’) and accumulation of DILP2 caused by lowering *edem1* levels in the fat body was rescued by *InR*^*A1325D*^ (Fig S1E).

Thus, blocking *edem1* in the fat body reduced systemic insulin signalling, which led to metabolic phenotypes. The rescue of phenotypes caused by lowering *edem1* levels by the constitutively active form of *InR* could be due to an increase in insulin signalling in the fat body cells or could be due to an indirect effect at the level of IPCs. These experiments confirmed that Edem1 function in the fat body is crucial to maintain systemic insulin signalling and metabolic homeostasis.

### Fat body–derived signals are involved in Edem1-mediated regulation of IPCs

*Drosophila* fat body controls IPC function with the aid of a set of humoral factors, which relays the nutritional status of the organism to the IPCs. The fat body–derived signals (FDSs) control DILP release from the IPCs into the hemolymph leading to effects on growth and maintenance of metabolic balance. In addition, changes in *dilp* gene expression have also been reported in response to fat body–derived signals. We next investigated whether blocking Edem1 led to changes in the levels of FDSs and thereby the function of IPCs.

**Figure S2.**
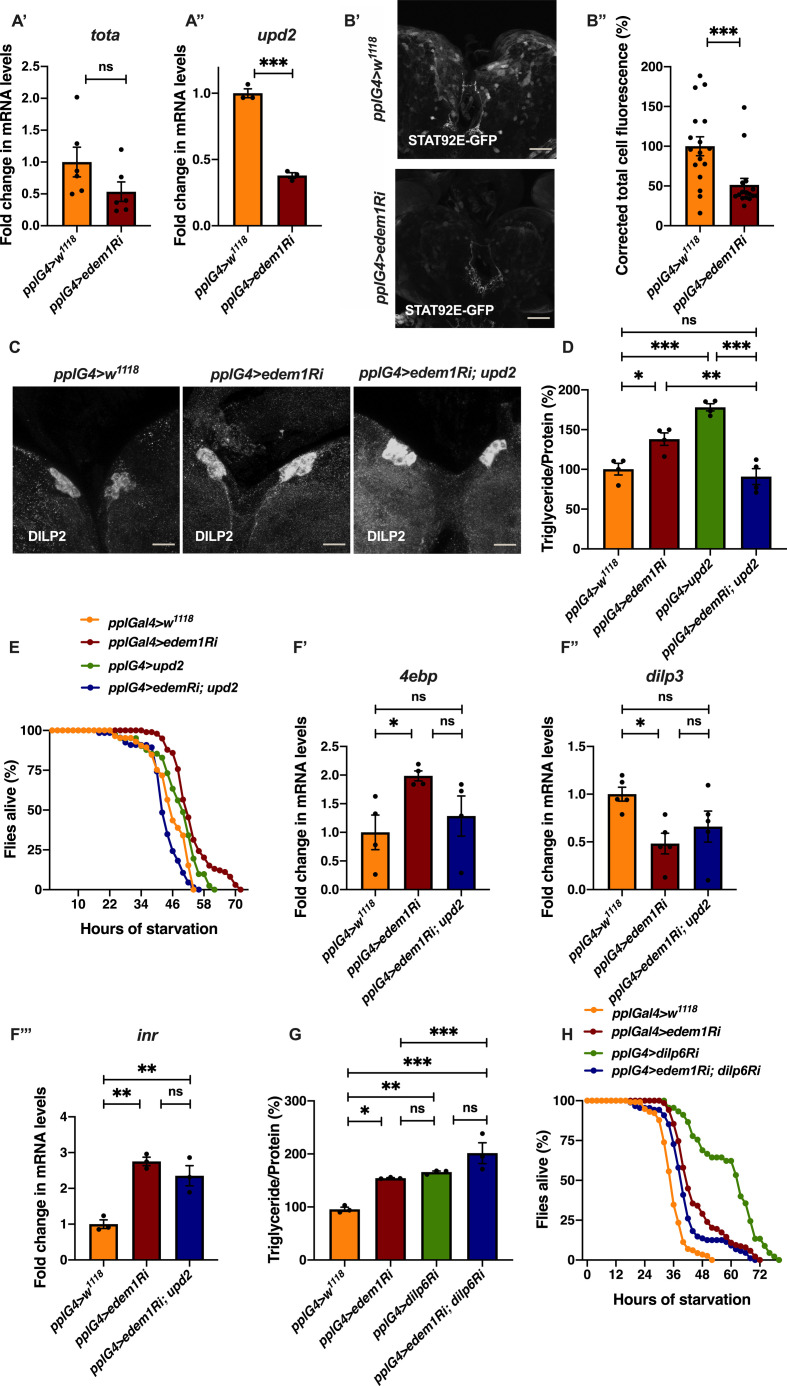
Co-expression of *upd2* but not *dilp6*-RNAi partially rescued the metabolic phenotypes. **(A)** Down-regulation of *edem1* in the larval fat body led to decrease in *totA* mRNA levels (A’). Data are shown as fold change in mRNA levels, values are normalised to *pplGal4>w*^*1118*^ and fold change in *pplGal4>UAS-edem1-RNAi* is shown (independent biological replicates = 6. *P*-value between control and *UAS-edem1-RNAi* is 0.0649 [Mann–Whitney test]). **(A’’)** Down-regulation of edem1 in the larval fat body led to decrease in upd2 mRNA levels (A’’). Data are shown as fold change in mRNA levels, values are normalised to *pplGal4>w*^*1118*^, and fold change in *pplGal4>UAS-edem1-RNAi* is shown (independent biological replicates = 6. *P*-value between control and *UAS-edem1-RNAi* is 0.0003 [Welch’s *t* test]). **(B)** Down-regulation of *edem1* in the larval fat body led to decrease in STAT92E-GFP expression (B’). Shown are the representative images of anti-GFP antibody staining in larval brains of *pplGal4>w*^*1118*^ (independent biological replicates = 17) and *pplGal4>UAS-edem1-RNAi* (independent biological replicates = 16). **(B’’)** Corrected total cell fluorescence values are normalised to *pplGal4>w*^*1118*^ and fold change in *pplGal4>UAS-edem1-RNAi* is shown (*P*-value between control and *UAS-edem1-RNAi* is 0.0017 [Mann–Whitney test]) (Scales: 35 µm). **(C)** DILP2 levels in the insulin-producing cells in response to *edem1-RNAi* were not rescued by expressing *UAS-upd2-EGFP* in the fat body (Fig S1D’). Shown are representative images of anti-DILP2 antibody staining in larval brains of *pplGal4>w*^*1118*^, *pplGal4>UAS-edem1-RNAi* and *pplGal4>UAS-edem1-RNAi*; *UAS-upd2-EGFP* (Scales: 25 µm). **(D)** Overexpression of Upd2 in the fat body rescued enhanced stored fat levels caused by *edem1*-RNAi. Data are shown as % ratio of triglyceride to total protein levels, values are normalised to *pplGal4>w*^*1118*^, and fold change in *pplGal4>UAS-edem1-RNAi*, *pplGal4>UAS-upd2-EGFP* and *pplGal4>UAS-edem1-RNAi*; *UAS-upd2-EGFP* is shown (independent biological replicates = 4, *P*-value between control and *UAS-edem1-RNAi* is 0.001, *P*-value between control and *UAS-upd2-EGFP* is <0.001, *P*-value between *UAS-edem1-RNAi* and *UAS-edem1-RNAi*, *UAS-upd2-EGFP* is 0.0002, *P*-value between *UAS-edem1-RNAi* and *UAS-upd2-EGFP* is 0.0370, *P*-value between *UAS-upd2-EGFP* and *UAS-edem1-RNAi*, *UAS-upd2-EGFP* is <0.001 and *P*-value between control and *UAS-edem1-RNAi*, *UAS-upd2-EGFP* is >0.9999 [Kruskal–Wallis test followed by Dunn’s post hoc test]). **(E)** Overexpression of Upd2 in the fat body rescued increased starvation resistance caused by *edem1-RNAi*. Data are shown as percentage of flies which were alive at various time points of starvation in the following genotypes—*pplGal4>w*^*1118*^, *pplGal4>UAS-edem1-RNAi*, *pplGal4>UAS-upd2-EGFP*, and *pplGal4>UAS-edem1-RNAi*; *UAS-upd2-EGFP* (independent biological replicates = 3, number of flies used for control is 85, for *pplGal4>UAS-edem1-RNAi* is 99, for *pplGal4> UAS-upd2-EGFP* is 41 and for *pplGal4> UAS-edem1-RNAi*, *UAS-upd2-EGFP* is 66. *P*-value between control and *UAS-edem1-RNAi* is <0.001, *P*-value between control and *UAS-upd2- EGFP* is <0.001, *P*-value between *UAS-edem1-RNAi* and *UAS-edem1-RNAi*, *UAS-upd2-EGFP* is <0.001, *P*-value between *UAS-edem1-RNAi* and *UAS-upd2-EGFP* is 0.0073, *P*-value between *UAS-upd2-EGFP* and *UAS-edem1-RNAi*, *UAS-upd2-EGFP* is <0.001 and *P*-value between control and *UAS-edem1-RNAi*, *UAS-upd2-EGFP* is 0.003 [log-rank test], Wald test = 9.01 on df = 1, *P* = 0.003 [Cox proportional hazard analysis]). **(F)** Increase in *4ebp* and *inr* and decrease in *dilp3* in response to *edem1*-RNAi was partially rescued by expressing *UAS-upd2-EGFP* in the fat body (F). Data are shown as fold change in mRNA levels, values are normalised to *pplGal4>w*^*1118*^ and fold change in *pplGal4>UAS-edem1-RNAi* and *pplGal4> UAS-edem1-RNAi*; *UAS-upd2-EGFP* is shown (independent biological replicates = 4. *P*-value between control and *UAS-edem1-RNAi* is 0.0277 for *4ebp*, 0.0155 for *dilp3*, 0.0015 for *inr*. *P*-value between control and *UAS-edem1-RNAi*; *UAS-upd2-EGFP* is >0.9999 for *4ebp*, 0.2305 for *dilp3*, 0.0055 for *inr*. *P*-value between *UAS-edem1-RNAi* and *UAS-edem1-RNAi*; *UAS-upd2-EGFP* is 0.1484 for *4ebp*, 0.9144 for *dilp3*, 0.3580 for *inr* [Kruskal–Wallis test followed by Dunn’s post hoc test for *4ebp* and *dilp3*, Ordinary one-way ANOVA followed by Tukey’s post hoc test for *inr*]). **(G)** Co-expression of *UAS-dilp6-RNAi* in the fat body did not rescue enhanced stored fat levels caused by *edem1-RNAi*. Data are shown as % ratio of triglyceride to total protein levels, values are normalised to *pplGal4>w*^*1118*^ and fold change in *pplGal4>UAS-edem1-RNAi*, *pplGal4> UAS-dilp6-RNAi* and *pplGal4> UAS-edem1-RNAi*; *UAS-dilp6-RNAi* is shown (independent biological replicates = 3, *P*-value between control and *UAS-edem1-RNAi* is 0.0061, *P*-value between control and *UAS-dilp6-RNAi* is 0.0013, *P*-value between *UAS-edem1-RNAi* and *UAS-edem1-RNAi*, *UAS-dilp6-RNAi* is 0.0019, *P*-value between *UAS-edem1-RNAi* and *UAS-dilp6-RNAi* is >0.9999, *P*-value between *UAS-dilp6-RNAi* and *UAS-edem1-RNAi*, *UAS-dilp6-RNAi* is >0.9999 and *P*-value between control and *UAS-edem1-RNAi*, *UAS-dilp6-RNAi* is <0.001 [Kruskal–Wallis test followed by Dunn’s post hoc test]). **(H)** Co-expression of *UAS-dilp6-RNAi* in the fat body did not rescue increased starvation resistance caused by *edem1-RNAi*. Data are shown as percentage of flies which were alive at various time points of starvation in the following genotypes—*pplGal4>w*^*1118*^, *pplGal4>UAS-edem1-RNAi*, *pplGal4>UAS-dilp6-RNAi*, *and pplGal4> UAS-edem1-RNAi*; *UAS-dilp6-RNAi* (independent biological replicates = 3, number of flies used for control is 115, for *pplGal4>UAS-edem1-RNAi* is 138, for *pplGal4>UAS-dilp6-RNAi* is 45, and for *pplGal4> UAS-edem1-RNAi*, *UAS-dilp6-RNAi* is 88. *P*-value between control and *UAS-edem1-RNAi* is <0.001, *P*-value between control and *UAS-dilp6-RNAi* is <0.001, *P*-value between *UAS-edem1-RNAi* and *UAS-edem1-RNAi*, *UAS-dilp6-RNAi* is 0.0035, *P*-value between *UAS-edem1-RNAi* and *UAS-dilp6-RNAi* is <0.001, *P*-value between *UAS-dilp6-RNAi* and *UAS-edem1-RNAi*, *UAS-dilp6-RNAi* is <0.001 and *P*-value between control and *UAS-edem1-RNAi*, *UAS-dilp6-RNAi* is <0.001 [log-rank test], Wald test = 33.26 on df = 1, *P* < 0.001 [Cox proportional hazard analysis]). **(A, B’’, D, F, G)** (*P*-value *<0.05; ** <0.01, *** <0.001; Data information: In [A, B’’, D, F, G], data are presented as mean ± SEM). Source data are available for this figure.

**Figure 3.**
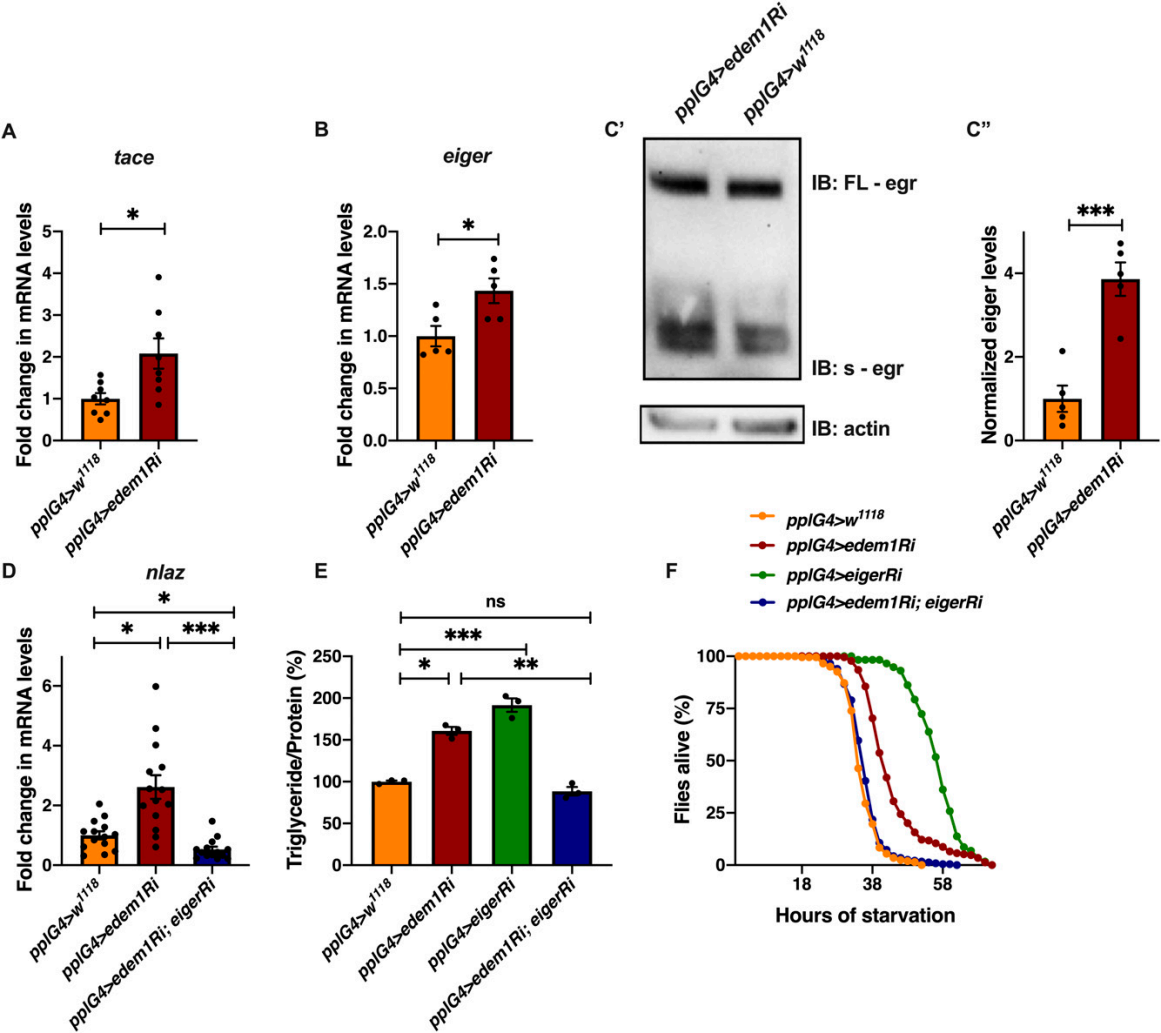
Reduction in *edem1* levels in the fat body enhanced JNK signalling. **(A)** Blocking *edem1* expression using RNAi in the fat body led to an increase in mRNA levels of *tace*. Data are shown as fold change in mRNA levels in third instar larvae, values are normalised to *pplGal4>w*^*1118*^ and fold change in *pplGal4>UAS-edem1-RNAi* is shown (independent biological replicates = 8, *P*-value between control and *UAS-edem1-RNAi* is 0.0211 [Welch’s *t* test]). **(B)** Blocking *edem1* expression using RNAi in the fat body led to an increase in mRNA levels of *eiger*. Data are shown as fold change in mRNA levels in third instar larvae, values are normalised to *pplGal4>w*^*1118*^ and fold change in *pplGal4>UAS-edem1-RNAi* is shown (independent biological replicates = 5, *P*-value between control and *UAS-edem1-RNAi* is 0.0226 [Welch’s *t* test]). **(C)** Blocking *edem1* expression using RNAi in the fat body led to an increase in the circulating levels of *eiger* in 5-d-old male flies (C’). Data are shown as fold change in normalised soluble eiger levels (C’’), values are normalised to *pplGal4>w*^*1118*^, and fold change in *pplGal4>UAS-edem1-RNAi* is shown (independent biological replicates = 5, *P*-value between control and *UAS-edem1-RNAi* is 0.0006 [Welch’s *t* test]). **(D)** Expression of *eiger*-RNAi in the fat body rescued increase in *nlaz* mRNA levels caused by blocking *edem1* levels in the fat body, and data are shown as fold change in mRNA levels. Data are shown as fold change in mRNA levels in third instar larvae, values are normalised to *pplGal4>w*^*1118*^ and fold change in *pplGal4>UAS-edem1-RNAi*, *pplGal4>UAS-eiger-RNAi* and *pplGal4>UAS-edem1-RNAi*; *UAS-eiger-RNAi* is shown (independent biological replicates = 14. *P*-value between control and *UAS-edem1-RNAi* is 0.0171, *P*-value between *UAS-edem1-RNAi* and *UAS-edem1-RNAi*; *UAS-eiger-RNAi* is <0.001 and between control and *UAS-edem1-RNAi*; *UAS-eiger-RNAi* is 0.1085 [Kruskal–Wallis test followed by Dunn’s post hoc test]). **(E)** Expression of *eiger*-RNAi in the fat body rescued increase in triglyceride caused by blocking *edem1* levels in the fat body. Data are shown as % ratio of triglyceride to total protein levels in 5-d-old male flies, normalised to 100% in *pplGal4>w*^*1118*^ (control), and changes in experimental conditions *pplGal4>UAS-edem1-RNAi* and *pplGal4>UAS-edem1-RNAi*; *UAS-eiger-RNAi* is shown (independent biological replicates = 3, *P*-value between control and *UAS-edem1-RNAi* is 0.0052, *P*-value between control and *UAS-eiger-RNAi* is <0.001, *P*-value between *UAS-edem1-RNAi* and *UAS-edem1-RNAi*, *UAS-eiger-RNAi* is <0.001, *P*-value between *UAS-edem1-RNAi* and *UAS-eiger-RNAi* is 0.8315, *P*-value between *UAS-eiger-RNAi* and *UAS-edem1-RNAi*, *UAS-eiger-RNAi* is <0.001 and *P*-value between control and *UAS-edem1-RNAi*, *UAS-eiger-RNAi* is >0.9999 [Kruskal–Wallis test followed by Dunn’s post hoc test]). **(F)** Enhanced starvation resistance shown as percentage of flies (5 d old) which were alive at various time points of starvation in the following genotypes—*pplGal4>w*^*1118*^, *pplGal4>UAS-edem1-RNAi*, and *pplGal4> UAS-edem1-RNAi*; *UAS-eiger-RNAi*—is shown (independent biological replicates = 3, number of flies used for control is 203, for *pplGal4>UAS-edem1-RNAi* is 229, for *pplGal4>UAS*-*eiger-RNAi* is 58 and for *pplGal4> UAS-edem1-RNAi*, *UAS*-*Ieiger-RNAi* is 233. *P*-value between control and *UAS-edem1-RNAi* is <0.001, *P*-value between control and *UAS-eiger-RNAi* is <0.001, *P*-value between *UAS-edem1-RNAi* and *UAS-edem1-RNAi*, *UAS-eiger-RNAi* is <0.001, *P*-value between *UAS-edem1-RNAi* and *UAS-eiger-RNAi* is <0.001, *P*-value between *UAS-eiger-RNAi* and *UAS-edem1-RNAi*, *UAS-eiger-RNAi* is <0.001 and *P*-value between control and *UAS-edem1-RNAi*, *UAS-eiger-RNAi* is 0.0459 [log-rank test], Wald test = 0.09 on df = 1, *P* = 0.8 [Cox proportional hazard analysis]). **(A, B, C’’, D, E)** (*P*-value *<0.05; ** <0.01, *** <0.001; Data information: In [A, B, C’’, D, E], data are presented as mean ± SEM). Source data are available for this figure.

To test the role of FDSs in *edem1* knock down phenotypes, we measured the levels or activity of various FDSs. We saw an increase in the transcript levels of *dilp6* in response to knock down of *edem1* in the fat body (Fig 2A’). Next, we measured the mRNA levels of *upd2*, *totA*, and the levels of STAT92E-GFP as readouts for activity of JAK-STAT pathway, a cell signalling pathway activated by Upd2, an FDS reported to regulate IPC functions (Rajan & Perrimon, 2013). Blocking *edem1* expression in the fat body led to a decrease in *totA* and *upd2* mRNA levels (Fig S2A’ and A’’). In addition, STAT92E-GFP expression in the brain was found to be low in response to reduced *edem1* in the fat body (Fig S2B’ and B’’). We also measured Drosophila TNFα Eiger levels, another FDS that acts on IPCs through its receptor Grindelwald, and activation of downstream JNK signalling by Eiger. The transcript levels of *tace*, the TNFα converting enzyme encoding gene, *eiger* and *nlaz*, a key target of JNK signalling were measured (Hull-Thompson et al, 2009; Pasco & Leopold, 2012; Agrawal et al, 2016), and the levels of these genes were found to be increased in response to *edem1*-RNAi (Fig 3A, B, and D). The cleaved form of Eiger protein (s-Egr) in the whole-body extracts was also found to be higher in *edem1*-RNAi (Fig 3C’ and C’’), which confirmed enhanced levels of active form of Eiger released by blocking Edem1 function in the fat body. Eiger and Dilp6 are considered to be negative regulators of IPC function, whereas Upd2 is expected to activate IPCs (Bai et al, 2012; Rajan & Perrimon, 2013; Agrawal et al, 2016), and the gene expression changes observed here suggested that these FDSs might mediate the effects of *edem1* knock down on insulin signalling.

### Eiger is involved in Edem1-mediated regulation of IPC

**Figure 4.**
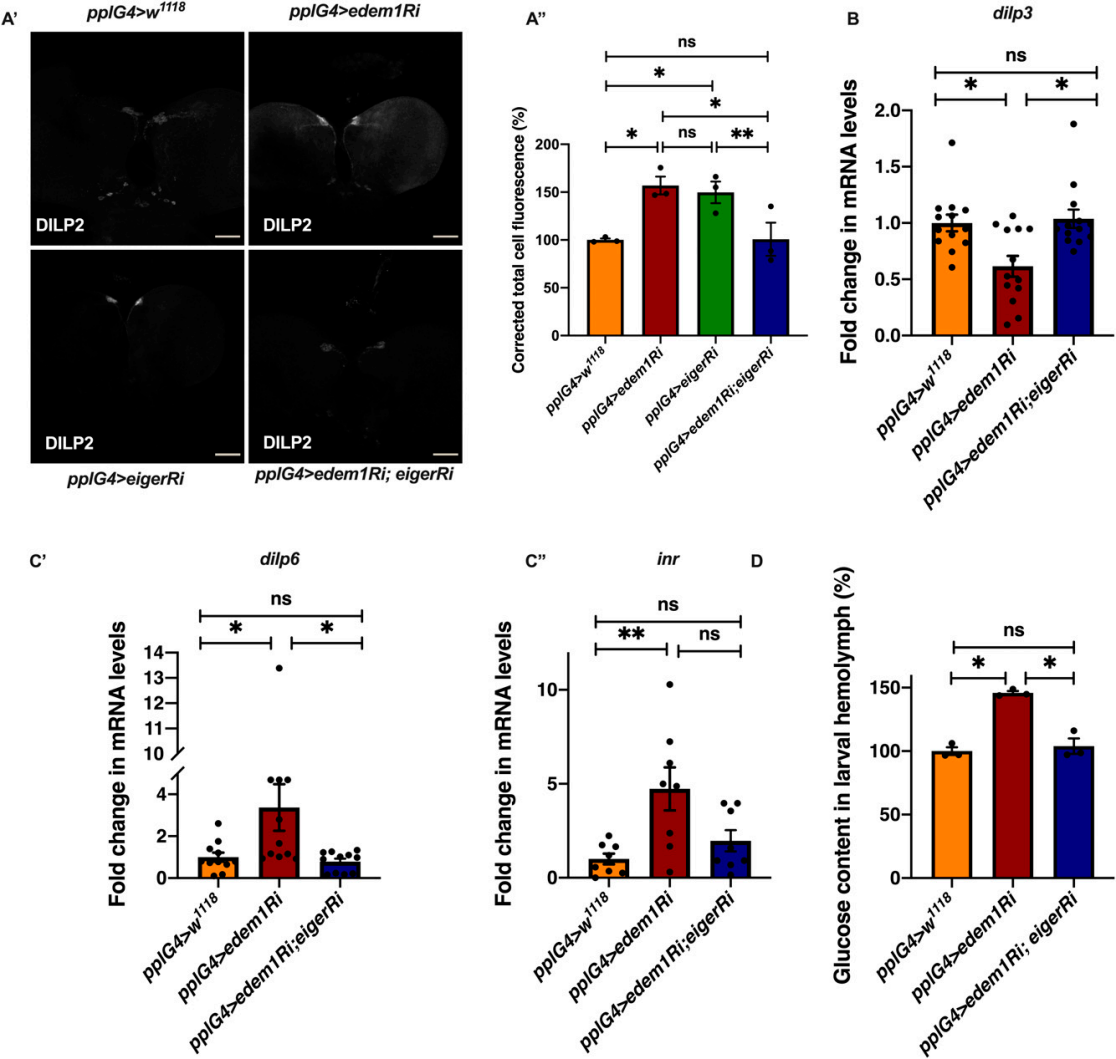
Knock down of *eiger* in the fat body rescued the metabolic phenotypes. **(A)** DILP2 levels in the insulin-producing cells in response to *edem1*-RNAi was rescued by reducing *eiger* in the fat body (A’). Shown are representative images of anti-DILP2 antibody staining in larval brains of *pplGal4>w*^*1118*^ (independent biological replicates = 15); *pplGal4>UAS-edem1-RNAi* (independent biological replicates = 14); *pplGal4>UAS-eiger-RNAi* (independent biological replicates = 19) and *pplGal4> UAS-edem1-RNAi*; *UAS-eiger-RNAi* (independent biological replicates = 9). Corrected total cell fluorescence values are normalised to *pplGal4>w*^*1118*^ and fold change in *pplGal4>UAS-edem1-RNAi*, *pplGal4>UAS-eiger-RNAi* and *pplGal4> UAS-edem1-RNAi*; *UAS-eiger-RNAi* is shown in (A’’) (*P*-value between control and *UAS-edem1-RNAi* is 0.0444, *P*-value between control and *UAS-eiger-RNAi* is 0.0270, *P*-value between *UAS-edem1-RNAi* and *UAS-eiger-RNAi* is 0.9967 *P*-value between control and *UAS-edem1-RNAi*; *UAS-eiger-RNAi* is >0.9999, *P*-value between *UAS-edem1-RNAi* and *UAS-edem1-RNAi*; *UAS-eiger-RNAi* is 0.0195 and *P*-value between *UAS-eiger-RNAi* and *UAS-edem1-RNAi*; *UAS-eiger-RNAi* is 0.0072 [Kruskal–Wallis test followed by Dunn’s post hoc test]) (scales: 50 µm). **(B)** Reduction of *dilp3* mRNA levels, in response to *edem1*-RNAi was rescued by reducing *eiger* in the fat body, data is shown as fold change in mRNA levels in third instar larvae, values are normalised to *pplGal4>w*^*1118*^ fold change in *pplGal4>UAS-edem1-RNAi* and *pplGal4> UAS-edem1-RNAi*; *UAS-eiger-RNAi* is shown (independent biological replicates = 13. *P*-value between control and *UAS-edem1-RNAi* is 0.0395, between control and *UAS-edem1-RNAi*; *UAS-eiger-RNAi* is >0.9999 and between *UAS-edem1-RNAi* and *UAS-edem1-RNAi*; *UAS-eiger-RNAi* is 0.0253 [Kruskal–Wallis test followed by Dunn’s post hoc test]). **(C)** Increase in *dilp6*, in response to *edem1*-RNAi was rescued by reducing *eiger* in the fat body (C’) and *inr* (C’’). Data are shown as fold change in mRNA levels in third instar larvae, values are normalised to *pplGal4>w*^*1118*^ and fold change in *pplGal4>UAS-edem1-RNAi* and *pplGal4> UAS-edem1-RNAi*; *UAS-eiger-RNAi* is shown (independent biological replicates = 11 for *dilp6* and independent biological replicates = 8 *for inr*. *P*-value between control and *UAS-edem1-RNAi* is 0.0392 for *dilp6* and 0.0194 for *inr*. *P*-value between control and *UAS-edem1-RNAi*; *UAS-eiger-RNAi* is >0.9999 *for dilp6* and >0.9999 for *inr*, *P*-value between *UAS-edem1-RNAi* and *UAS-edem1-RNAi*; *UAS-eiger-RNAi* is 0.0296 for *dilp6* and 0.1976 for *inr* [Kruskal–Wallis test followed by Dunn’s post hoc test]). **(D)** Increase in Glucose levels in the hemolymph in response to *edem1*-RNAi was rescued by reducing *eiger* in the fat body. Data are shown as % of glucose levels in the larval hemolymph, normalised to 100% in *pplGal4>w*^*1118*^ (control) and changes in experimental conditions *pplGal4>UAS-edem1-RNAi* and *pplGal4> UAS-edem1-RNAi*; *UAS-eiger-RNAi is shown* (independent biological replicates = 3, *P*-value between control and *UAS-edem1-RNAi* is 0.0105, *P*-value between control and *UAS-edem1-RNAi*; *UAS-eiger-RNAi* is >0.9999, *P*-value between *edem1-RNAi* and *UAS-edem1-RNAi*; *UAS-eiger-RNAi* is 0.0175 [Kruskal–Wallis test followed by Dunn’s post hoc test]). **(A’’, B, C, D)** (*P*-value *<0.05; ** <0.01, *** <0.001; Data information: In [A’’, B, C, D], data are presented as mean ± SEM). Source data are available for this figure.

To identify the FDS(s) involved in mediating the metabolic phenotypes observed by blocking *edem1* levels, we expressed *eiger*-RNAi, *dilp6*-RNAi or *upd2*, together with *edem1*-RNAi in the fat body. Down-regulation of *eiger* mRNA and overexpression of *upd2* rescued *edem1* knock down phenotypes of lipid stores and starvation resistance; however, there were no effects with *dilp6*-RNAi (Figs 3E and F and S2D, E, G, and H). This suggested that either Upd2 or Eiger could be regulated by Edem1 and manage IPC function. We did not see any significant changes to DILP2 levels in the IPCs, *dilp3* mRNA levels and insulin target genes by overexpression of *upd2* in fat body that express *edem1*-RNAi (Fig S2C and F). However, when we down-regulated *eiger* mRNA in *edem1*-RNAi expressing fat body, we observed a reduction in DILP2 puncta in the IPCs seen in response to *edem1*-RNAi expression in the fat body (Fig 4A’ and A’’). In addition, transcript levels of *dilp3* (Fig 4B) and insulin target genes *dilp6* (Fig 4C’) *and inr* (Fig 4C’’) were restored by reducing Eiger levels in the *edem1*-RNAi background. Furthermore, the increase in glucose levels in the hemolymph seen in response to blocking *edem1* was suppressed by co-expression of *eiger*-RNAi (Fig 4D). Thus, Edem1-mediated regulation of Eiger is crucial for managing insulin levels and nutrient homeostasis, by acting on the IPCs. Whereas, the Edem1-mediated regulation of Upd2 function also manages nutrient homeostasis, but does not do so by acting at the level of insulin signalling, suggesting an IPC-insulin signalling independent regulation of metabolic status by Edem1.

The fat body–derived cytokine Eiger is an upstream activator of c-Jun N-terminal kinase (JNK) pathway in flies and previous studies have shown that JNK signalling extends life span and limits growth by antagonizing cellular and organism-wide responses to insulin signalling (Hirosumi et al, 2002; Oh et al, 2005; Wang et al, 2005; Andersen et al, 2015; Agrawal et al, 2016). The increase in *nlaz* transcript levels in response to blocking *edem1* levels in the fat body was rescued completely by the co-expression of *eiger*-RNAi, showing that the increase in JNK signalling in response to blocking *edem1* expression in the fat body is due to enhanced Eiger levels (Fig 3D). These experiments confirm that Edem1 function in the fat body regulates Eiger activity and JNK signalling.

**Figure 5.**
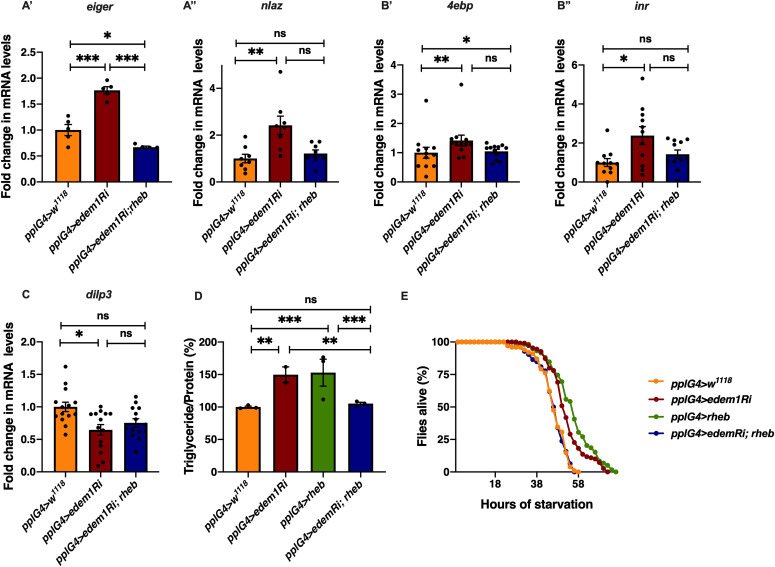
Activating target of rapamycin signalling rescued *edem1*-mediated phenotypes. **(A)** Increase in mRNA levels of *eiger* (A’) and *nlaz* (A’’) in response to blocking *edem1* expression was rescued by co-expression of *UAS-rheb*. Data are shown as fold change in mRNA levels in third instar larvae, values are normalised to *pplGal4>w*^*1118*^ and fold change in *pplGal4>UAS-edem1-RNAi* and *pplGal4> UAS-edem1-RNAi*; *UAS-rheb* is shown (independent biological replicates = 5 for *eiger* and 8 for *nlaz*. *P*-value between control and *UAS-edem1-RNAi* is <0.0001, *P*-value between *UAS-edem1-RNAi* and *UAS-edem1-RNAi*; *UAS-rheb* is <0.0001, *P*-value between control and *UAS-edem1-RNAi*; *UAS-rheb* is 0.0228 for *eiger* [ordinary one-way ANOVA followed by Tukey’s post hoc test]. *P*-value between control and *UAS-edem1-RNAi* is 0.0039, *P*-value between *UAS-edem1-RNAi* and *UAS-edem1-RNAi*; *UAS-rheb* is 0.0486, *P*-value between control and *UAS-edem1-RNAi*; *UAS-rheb* is >0.9999 for *nlaz* [Kruskal–Wallis test followed by Dunn’s post hoc test]). **(B)** Increase in mRNA levels of *4ebp* (B’) and *inr* (B’’) in response to blocking *edem1* expression was alleviated by co-expression of *UAS-rheb*. Data are shown as fold change in mRNA levels in third instar larvae, values are normalised to *pplGal4>w*^*1118*^ and fold change in *pplGal4>UAS-edem1-RNAi* and *pplGal4> UAS-edem1-RNAi*; *UAS-rheb* is shown (independent biological replicates = 12 for *4ebp*. *P*-value between control and *edem1*-RNAi is 0.0085, *P*-value between *UAS-edem1-RNAi* and *UAS-edem1-RNAi*; *UAS-rheb* is 0.1143, *P*-value between control and *UAS-edem1-RNAi*; *UAS-rheb* is >0.9999 [Kruskal–Wallis test followed by Dunn’s post hoc test]; independent biological replicates = 11 for *inr*. *P*-value between control and *UAS-edem1-RNAi* is 0.0406, *P*-value between *UAS-edem1-RNAi* and *UAS-edem1-RNAi*; *UAS-rheb* is 0.7277, *P*-value between control and *UAS-edem1-RNAi*; *UAS-rheb* is 0.5799 [Kruskal–Wallis test followed by Dunn’s post hoc test]). **(C)** Decrease in mRNA levels of *dilp3* in response to blocking *edem1* expression was rescued by co-expression of *UAS-rheb*. Data are shown as fold change in mRNA levels in third instar larvae, values are normalised to *pplGal4>w*^*1118*^ and fold change in *pplGal4>UAS-edem1-RNAi* and *pplGal4> UAS-edem1-RNAi*; *UAS-rheb* is shown (independent biological replicates = 14. *P*-value between control and *UAS-edem1-RNAi* is 0.0231, *P*-value between *UAS-edem1-RNAi* and *UAS-edem1-RNAi*; *UAS-rheb* is >0.9999, *P*-value between control and *UAS-edem1-RNAi*; *UAS-rheb* is 0.1356 [Kruskal–Wallis test followed by Dunn’s post hoc test]). **(D)** Overexpression of *rheb* in the fat body rescued enhanced stored fat levels. Data are shown as % ratio of triglyceride to total protein levels, in 5-d-old male flies, values are normalised to *pplGal4>w*^*1118*^ and fold change in *pplGal4>UAS-edem1-RNAi* and *pplGal4> UAS-edem1-RNAi*; *UAS-rheb* is shown (independent biological replicates = 4, *P*-value between control and *UAS-edem1-RNAi* is 0.0003, *P*-value between control and *UAS-rheb* is <0.001, *P*-value between *UAS-edem1-RNAi* and *UAS-edem1-RNAi*, *UAS-rheb* is 0.0005, *P*-value between *UAS-edem1-RNAi* and *UAS-rheb* is 0.5184, *P*-value between *UAS-rheb* and *UAS-edem1-RNAi*, *UAS-rheb* is <0.001 and *P*-value between control and *UAS-edem1-RNAi*, *UAS-rheb* is >0.9999 [Kruskal–Wallis test followed by Dunn’s post hoc test]). **(E)** Overexpression of *rheb* in the fat body rescued increased starvation resistance. Data are shown as percentage of 5-d-old male flies which were alive at various time points of starvation in the following genotypes—*pplGal4>w*^*1118*^, *pplGal4>UAS-edem1-RNAi*, *pplGal4>UAS-rheb*, and *pplGal4> UAS-edem1-RNAi*; *UAS-rheb* (independent biological replicates = 5, number of flies used for control is 101, for *pplGal4>UAS-edem1-RNAi* is 110, for *pplGal4>UAS*-*rheb* is 59 and for *pplGal4> UAS-edem1-RNAi*, *UAS*-*rheb* is 126. *P*-value between control and *UAS-edem1-RNAi* is <0.0001, *P*-value between control and *UAS-edem1-RNAi* is <0.001, *P*-value between control and *UAS-rheb* is <0.001, *P*-value between *UAS-edem1-RNAi* and *UAS-edem1-RNAi*, *UAS-rheb* is <0.001, *P*-value between *UAS-edem1-RNAi* and *UAS-rheb* is 0.0247, *P*-value between *UAS-rheb* and *UAS-edem1-RNAi*, *UAS-rheb* is <0.001 and *P*-value between control and *UAS-edem1-RNAi*, *UAS-rheb* is 0.9628 [log-rank test], Wald test = 0.21 on df = 1, *P* = 0.6 [Cox proportional hazard analysis]). **(A, B, C, D)** (*P*-value *<0.05; ** <0.01, *** <0.001; Data information: In [A, B, C, D], data are presented as mean ± SEM). Source data are available for this figure.

**Figure 6.**
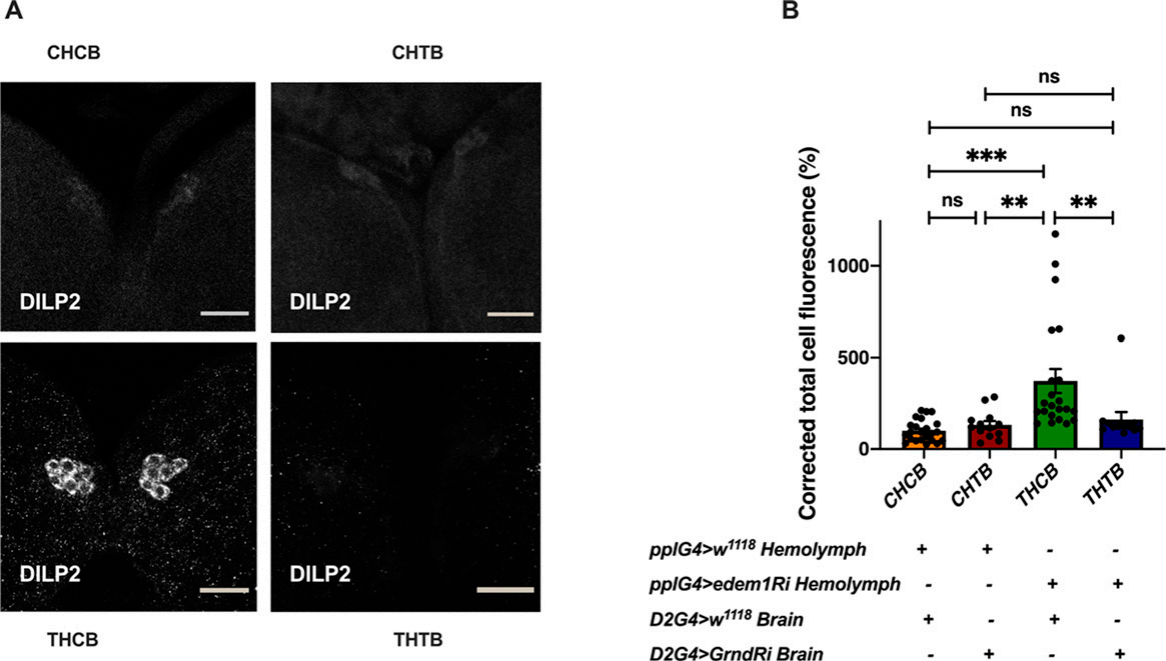
Blocking *grindelwald* in the insulin-producing cells (IPCs) rescued the increased accumulation of DILP2. **(A)** DILP2 accumulation in the IPCs in response to *edem1*-RNAi was rescued by blocking Eiger receptor *grnd* in the IPCs. Representative images of anti-DILP2 antibody staining in larval brains of *Dilp2Gal4>w*^*1118*^ treated with hemolymph from *pplGal4*>*w*^*1118*^ (independent biological replicates = 23) (CHCB), *Dilp2Gal4>UAS-grnd-RNAi* treated with hemolymph from *pplGal4*>*w*^*1118*^ (independent biological replicates = 13) (CHTB), *Dilp2Gal4>w*^*1118*^ incubated with hemolymph from *pplGal4>UAS-edem1-RNAi* (independent biological replicates = 22) (THCB) and *Dilp2Gal4>UAS-grnd-RNAi* incubated with hemolymph from *pplGal4>UAS-edem1-RNAi* (independent biological replicates = 12) (THTB) larvae are shown (scales: 30 µm). **(B)** Corrected total cell fluorescence values are normalised to CHCB and fold change in CHTB, THCB, and THTB are shown (*P*-value between CHCB and CHTB is >0.9999, *P*-value between CHCB and THCB is <0.001, *P*-value between CHCB and THTB is >0.9999, *P*-value between CHTB and THCB is 0.0018, *P*-value between CHTB and THTB is >0.9999 and *P*-value between THCB and THTB is 0.0085 [Kruskal–Wallis test followed by Dunn’s post hoc test]). **(B)** (*P*-value *<0.05; ** <0.01, *** <0.001; Data information: In [B], data are presented as mean ± SEM). Source data are available for this figure.

Next, we carried out experiments to confirm if regulation of Eiger by Edem1 is crucial for maintaining IPC function and metabolic homeostasis. Towards this we used two approaches: (i) we activated TOR signalling pathway in *edem1*-RNAi expressing fat body (Fig 5), as TOR signalling has been shown to block Eiger activation and (ii) we performed co-culture experiments by blocking the TNF receptor *grindelwald* in the IPCs (Fig 6). TOR signalling pathway regulates a plethora of cellular processes including cell growth, proliferation, cell survival, etc., depending on nutrient levels. Recently, TOR has been reported to repress *tace* transcription, which would in turn suppress the production of active Eiger from the fat body (Agrawal et al, 2016). Rheb (Ras homolog enriched in brain), a member of Ras superfamily of GTP binding proteins, activates TOR kinase and results in growth and regulation of metabolic pathways (Oldham et al, 2000; Garami et al, 2003; Saucedo, 2003; Oldham, 2011). Increase in the levels of *eiger* transcript levels and JNK pathway target *nlaz* in response to *edem1* down-regulation in the fat body was abrogated by overexpression of *rheb* (Fig 5A), which confirms that activating TOR signalling is sufficient to suppress JNK signalling possibly by the regulation of Eiger activity. Changes in the reduction in insulin signalling (Fig 5B), *dilp3* transcript levels (Fig 5C), excess fat levels (Fig 5D), and starvation resistance (Fig 5E) in response to blocking Edem1 levels were rescued by the overexpression of *rheb* in the fat body. This confirmed that increasing TOR activity and thereby reducing the activation of JNK levels was sufficient to rescue the systemic insulin signalling and the metabolic phenotypes such as fat storage and starvation sensitivity.

Next, we tried to show that Edem1 function in the fat body regulates Eiger activity on IPCs. In low-protein diet, the soluble form of Eiger binds to its receptor Grindelwald (Grnd) in the IPCs, thereby activate JNK signalling and suppress *dilp* transcript levels (Andersen et al, 2015; Agrawal et al, 2016). We performed ex vivo organ culture experiments, using hemolymph isolated from control larvae and larvae in which *edem1* expression in the fat body was blocked. Brains dissected from wild-type larvae and larvae with *grnd* knock down in the IPCs, were incubated with the hemolymph from the above-mentioned conditions. As expected, treatment of control larval brains with hemolymph from *edem1*-RNAi larvae (THCB) led to accumulation of DILP2 in the IPCs, when compared with hemolymph from control larvae (CHCB) (Fig 6A and B). We observed less DILP2 puncta in IPCs in response to blocking *grnd* (THTB) when compared with control IPCs treated with hemolymph from *edem1*-RNAi larvae (THCB) (Fig 6A and B). Blocking *grnd* did not change the levels of DILP2 in the IPCs as *IPC>grnd*-*RNAi* brains did not show any changes in DILP2 puncta when treated with hemolymph extracted from control larvae (CHTB). Thus, accumulation of DILP2 in the IPCs in response to blocking *edem1* levels in the larval fat body is due to Eiger activity on the IPCs through TNF receptor Grindelwald. Together these results confirm that Edem1 activity in the fat body regulates Eiger-mediated JNK signalling in the IPCs and manage systemic insulin signalling and metabolic status of flies.

### Reduction in *edem1* levels during starvation is crucial for survival

**Figure 7.**
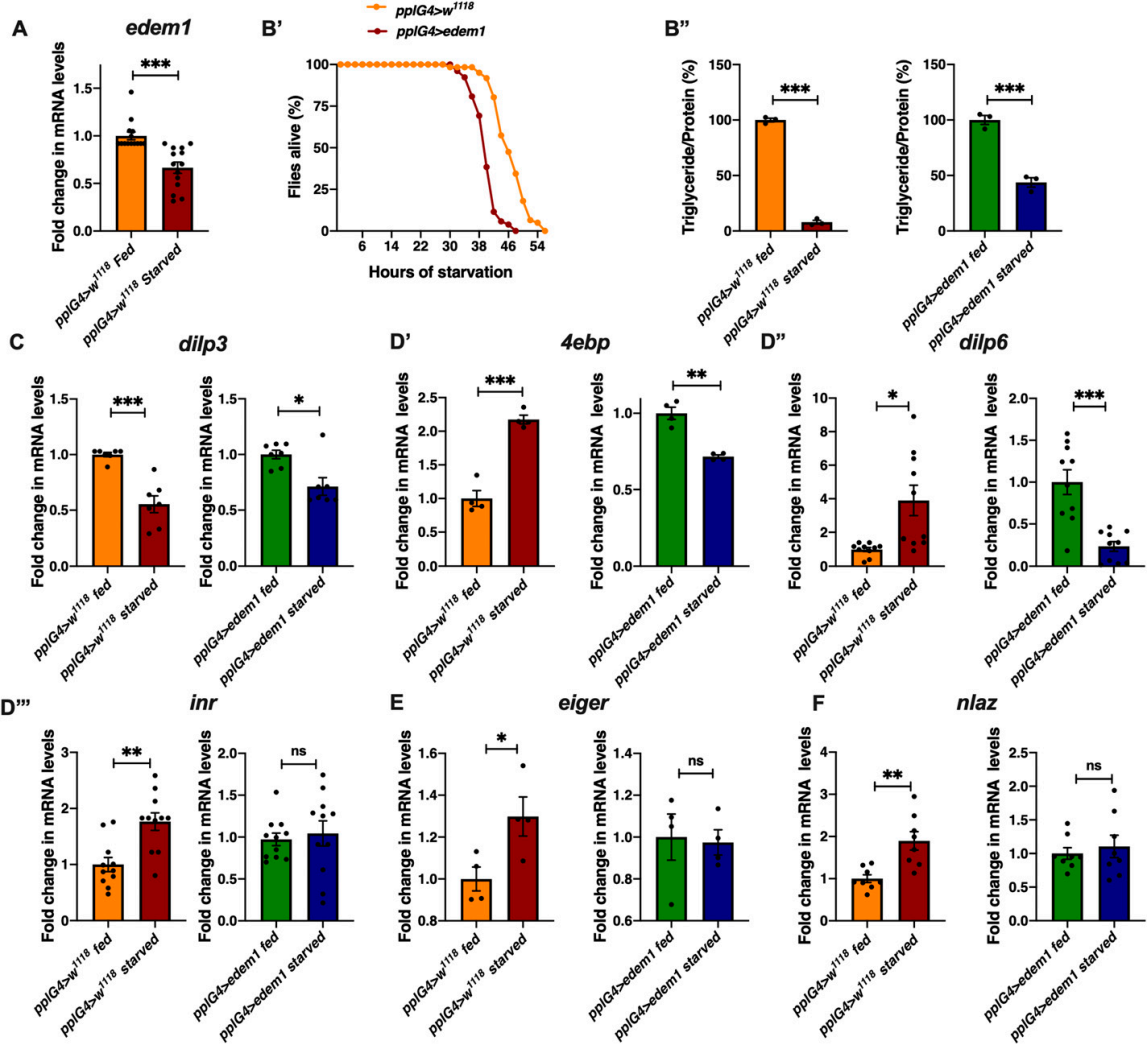
Reduction in *edem1* levels during starvation is crucial for survival. **(A)** Fold change in the mRNA levels of *edem1* upon starvation in *pplGal4>w*^*1118*^ larvae. Values are normalised to *pplGal4>w*^*1118*^ fed control and changes in the control starved are shown (independent biological replicates = 14, *P*-value between control fed and starved larvae is <0.0001 [Mann–Whitney test]). **(B)** Overexpression of *edem1* leads to enhanced sensitivity to starvation (B’), shown are percentage of male flies which were alive at various time points of starvation in the following genotypes—*pplGal4>w*^*1118*^ and *pplGal4>UAS-edem1* (independent biological replicates = 3, number of flies used for control is 74, for *pplGal4>UAS-edem1* is 69. *P*-value between control and *UAS-edem1* is <0.0001 [log-rank test], Wald test = 20.67 on df = 1, *P* < 0.001 [Cox proportional hazard analysis]). **(B’’)** shows percentage reduction in triglyceride levels upon starvation. Data are shown as % ratio of triglyceride to total protein levels, values are normalised to *pplGal4>w*^*1118*^ fed and fold change in *pplGal4>w*^*1118*^ starved is shown (left) and values are normalised to *pplGal4>UAS-edem1* fed and fold change in *pplGal4>UAS-edem1* starved is shown (right) (independent biological replicates = 3, *P*-value between *pplGal4>w*^*1118*^ fed and *pplGal4>w*^*1118*^ starved is <0.0001; *P*-value between *pplGal4>UAS-edem1* fed and *pplGal4>UAS-edem1* starved is <0.0001 Welch’s *t* test) **(C)** Overexpression of *edem1* in the fat body decreased the *dilp3* mRNA levels when subjected to starvation. Data are shown as fold change in mRNA levels, values are normalised to *pplGal4>w*^*1118*^ fed and fold change in *pplGal4>w*^*1118*^ starved is shown (left) and values are normalised to *pplGal4>UAS-edem1* fed and fold change in *pplGal4>UAS-edem1* starved is shown (right) (independent biological replicates = 7. *P*-value between control fed and starved is <0.001. *P*-value between *UAS-edem1* fed and *UAS-edem1* starved is 0.0251 [Mann–Whitney test]). **(D)** Overexpression of *edem1* in the fat body led to decreased levels of *4ebp* (D’), *dilp6* (D’’) and no change in *inr* (D’’’) when subjected to starvation. Data are shown as fold change in mRNA levels, values are normalised to *pplGal4>w*^*1118*^ fed and fold change in *pplGal4>w*^*1118*^ starved is shown (left) and values are normalised to *pplGal4>UAS-edem1* fed and fold change in *pplGal4>UAS-edem1* starved is shown (right) (independent biological replicates = 4 for *4ebp*, independent biological replicates = 10 for *dilp6* and independent biological replicates = 11 for *inr*. *P*-value between control fed and starved is <0.001 for *4ebp*, 0.0107 for *dilp6*, and 0.0012 for *inr*. *P*-value between *UAS-edem1* fed and *UAS-edem1* starved is 0.0039 for *4ebp*, <0.001 for *dilp6* and 0.6788 for *inr* [Welch’s *t* test]). **(E)** Overexpression of *edem1* in the fat body did not affect the levels of *eiger* mRNA when subjected to starvation. Data are shown as fold change in mRNA levels, values are normalised to *pplGal4>w*^*1118*^ fed and fold change in *pplGal4>w*^*1118*^ starved is shown (left) and values are normalised to *pplGal4>UAS-edem1* fed and fold change in *pplGal4>UAS-edem1* starved is shown (right) (independent biological replicates = 4. *P*-value between control fed and starved is 0.0417. *P*-value between *UAS-edem1* fed and *UAS-edem1* starved is 0.8486 [Welch’s *t* test]). **(F)** Overexpression of *edem1* in the fat body did not affect the levels of *nlaz* mRNA when subjected to starvation. Data are shown as fold change in mRNA levels, values are normalised to *pplGal4>w*^*1118*^ fed and fold change in *pplGal4>w*^*1118*^ starved is shown (left) and values are normalised to *pplGal4>UAS-edem1* fed and fold change in *pplGal4>UAS-edem1* starved is shown (right) (independent biological replicates = 8. *P*-value between control fed and starved is 0.004. *P*-value between *UAS-edem1* fed and *UAS-edem1* starved is 0.5887 [Welch’s *t* test]). **(A, B’’, C, D, E, F)** (*P*-value *<0.05; ** <0.01, *** <0.001; Data information: In [A, B’’, C, D, E, F], data are presented as mean ± SEM). Source data are available for this figure.

**Figure S3.**
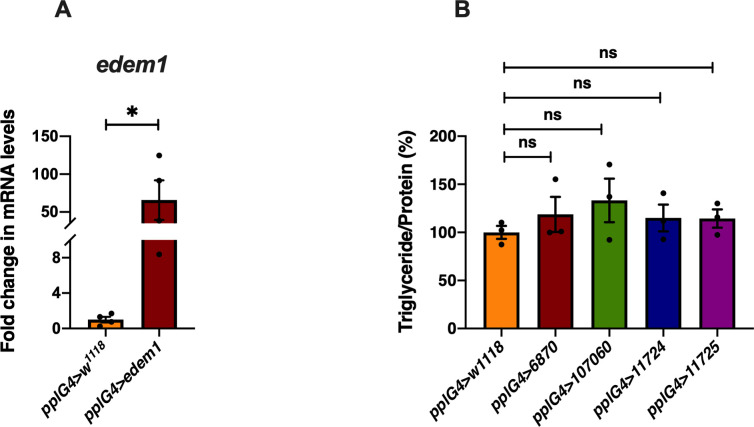
Fold change in *edem1* mRNA levels in response to edem1 overexpression in the fat body. **(A)** Overexpression of *edem1* in the fat body led to increased levels of *edem1* mRNA when compared with control. Data are normalised to *pplGal4>w*^*1118*^ and fold change in *pplGal4>UAS-edem1-RNAi* is shown (independent biological replicates = 4 *P*-value between control and *UAS-edem1-RNAi* is 0.0477 [unpaired *t* test]). **(B)** Blocking ER-associated degradation using RNAi against *sip3* and *herp* in the fat body did not affect the triglyceride levels 5-d-old flies. Data are shown as % ratio of triglyceride to total protein levels, normalised to 100% in *pplGal4>w*^*1118*^ (control) and experimental conditions *pplGal4>UAS-sip3-RNAi* (6870 and 107060) and *pplGal4>UAS-herp-RNAi* (11724 and 11725) (independent biological replicates = 3, *P*-value between control and *pplG4>6870*, *pplG4>107060*, *pplG4>11724*, and *pplG4>11725* is >0.9999 [Kruskal–Wallis test followed by Dunn’s post hoc test]). **(A, B)** (*P*-value *<0.05; ** <0.01, *** <0.001; Data information: In [A, B], data are presented as mean ± SEM). Source data are available for this figure.

Insulin signalling aids an organism to respond to changes in the nutrient environment by managing various biological functions. In response to nutrient deprivation insulin signalling is reduced, which would allow an organism to manage its energy stores and induce various hunger triggered behavioral responses (Britton et al, 2002; Arsic & Guerin, 2008; Erion & Sehgal, 2013). Our experiments show that lowering *edem1* levels improved survival against starvation by reducing insulin signalling (Figs 1C and 2E). Hence, we tested if *edem1* levels are lowered during nutrient deprivation, which may aid in better survival of flies by the reduction in insulin levels. Moreover, blocking *edem1* in the fat body in larvae was sufficient to enhance the appetite, similar to hunger induced responses observed in food deprived larvae (Fig 1G). As expected, in response to food depletion we observed a reduction in *edem1* mRNA levels (Fig 7A). To test whether reduction in *edem1* levels is essential for protection against starvation, we overexpressed *edem1* in the fat body in food deprived flies and checked their starvation responses. Overexpression of *edem1* in the fat body reduced the survival of flies during food deprivation (Figs 7B’ and S3A), confirming that the reduction in *edem1* levels is crucial for survival in response to nutrient depletion. Whereas overexpression of Edem1 did not affect fat stores in the adult flies, higher levels of Edem1 blocked utilisation of TGA in starved animals and caused enhanced mortality (Fig 7B). Thus, reduction in Edem1 during starvation facilitates mobilisation of stored fat, which aids in enhanced survival by meeting energy requirements during acute food deprivation. Higher Edem1 levels during starvation blocked the increase in *eiger* transcripts (Fig 7C), although the levels of the cleaved form of Eiger did not get affected either in fed or starved conditions. In response to acute starvation, JNK signalling is increased and the transcript levels of *dilp3* and insulin signalling are decreased (Fig 7C–F). These effects of starvation were abrogated by *edem1* overexpression in the fat body (Fig 7C–F). Meanwhile, overexpression of Edem1 in the fed conditions did not affect fat levels and insulin signalling. Thus, elevated Edem1 expression affects metabolic homeostasis and survival during starvation conditions. Here, we conclude that lowering of *edem1* transcripts in the fat body during starvation facilitates activation of Eiger and reduction in insulin signalling, which results in the enhanced survival of flies.

## Discussion

Fluctuations in nutrient levels would trigger organism-wide changes, which includes alterations to various metabolic pathways. Changes in the metabolic pathways would aid the organism in managing the growth and maintenance of nutrient stores according to the availability of food. Apart from these biochemical changes, hunger elicits stereotypic behavioral responses, which includes an enhanced urge to feed, increased foraging, acceptance of unpalatable food, etc. (Chouhan et al, 2017). Several of the crucial responses such as mobilisation of stored nutrients and enhanced urge to feed, which aids the organism to survive nutrient deprivation is triggered by the reduction in systemic insulin signalling (Britton et al, 2002; Rulifson et al, 2002; Broughton et al, 2005; Tettweiler et al, 2005; Arsic & Guerin, 2008; DiAngelo & Birnbaum, 2009; Kannan & Fridell, 2013). In *Drosophila*, IPCs respond to changes in the availability of food and modulate DILP levels under the control of the fat body, which acts as a nutrient sensor. Various fat body–derived signals act on IPCs directly or indirectly, and the regulation of these signals in response to changes in the nutrient status of *Drosophila* plays a key role in maintaining systemic insulin levels (Colombani et al, 2003; Géminard et al, 2009; Bai et al, 2012; Rajan & Perrimon, 2012; Ghosh & O’Connor, 2014; Agrawal et al, 2016; Delanoue et al, 2016; Droujinine & Perrimon, 2016; Koyama & Mirth, 2016; Sun et al, 2017). Here, we report a novel means by which the activity of a fat body–derived signal on IPCs is regulated.

**Figure 8.**
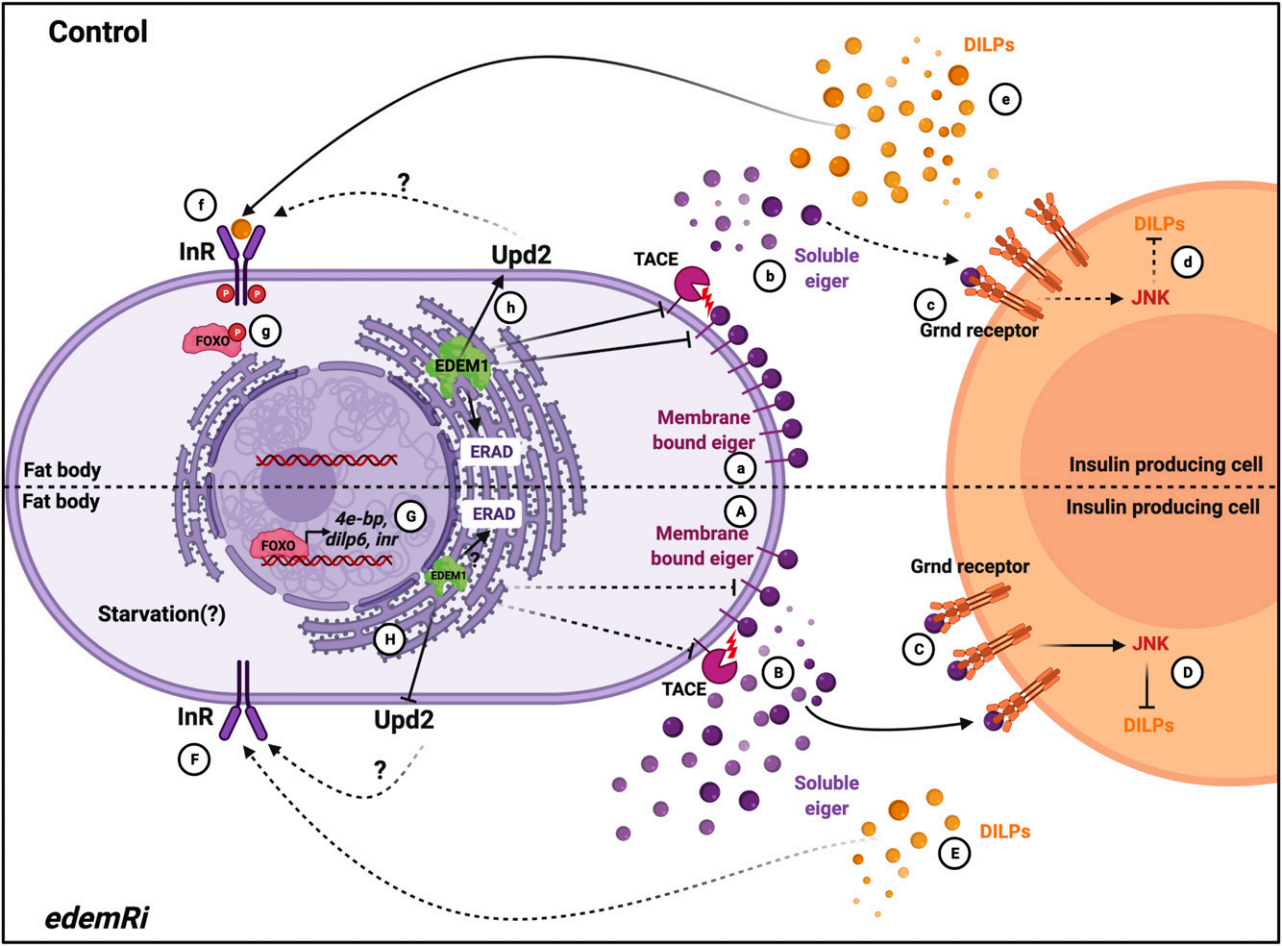
Working model. In control and fed conditions, Edem1 blocks *tace* and *eiger* gene expression (a) and inhibits Eiger release (b) and as JNK signalling is kept low in the insulin-producing cells (c). This maintains insulin signalling (d-g). In *edem1*Ri background, *tace* and *eiger* gene expression is increased (A) and leads to increased Eiger secretion into the hemolymph (B). Soluble Eiger binds to the Grnd receptors in the insulin-producing cells and activate JNK signalling which inhibits insulin signalling (C and D). Reduction in insulin signalling mediated by reduction in Edem1 in the fat body (E, F, G) aid in survival of flies. Edem maintains Upd2 levels in the organisms (h) which maintain the metabolic homeostasis, but in *edem1*Ri background Upd2 levels decrease (H) resulting in metabolic phenotypes (created with BioRender.com).

While investigating the mechanisms that function in the fat body to control *Drosophila* IPCs, we identified Edem1, an ER-resident protein involved in ERAD mediated protein quality control. Edem1 in the fat body maintains the activity of *Drosophila* TNFα Eiger (Fig 8A and B) and controls JNK signalling (Fig 8C), thereby promoting normal IPC function (Fig 8D), maintain systemic insulin signalling (Fig 8E–G) and metabolic homeostasis (Figs 3 and 4). Eiger is activated by TACE, which cleaves the transmembrane form of Eiger and releases a soluble active form of Eiger into the hemolymph (Agrawal et al, 2016). TOR kinase, a key nutrient sensor, has been reported to control *tace* transcript levels and thereby Eiger activation. During low-protein diets, because of reduced TOR signalling, fat body releases the soluble form of Eiger, which would act on IPCs and activate JNK signalling to regulate *dilp* gene expression. Here, we identify Edem1 as a regulator of Eiger through the control of *eiger* and *tace* gene expression (Fig 3). We also show that activation of TOR signalling blocked the effects of suppression of *edem1* levels in the fat body (Fig 5), substantiating the role of Edem1 in regulating Eiger activity. At the moment, it is not clear if the TOR pathway acts through Edem1 to regulate *eiger and tace* gene expression, thereby manage Eiger activity. More efforts are also needed to identify the exact molecular mechanism by which Edem1 regulates Eiger.

We also found that the levels of Upd2, another FDS, are regulated by Edem1 function in the fat body. The fact that Upd2OE in *edem1Ri* background does not rescue the DILP2 accumulation and mRNA levels of *dilp3* and other insulin targets suggests that Upd2 may act through another tissue unknown at the moment to regulate nutrient homeostasis. In support of this, we saw a decrease in *tota* in the whole larval RNA extracts and STAT92E-GFP levels in the entire brain in response to blocking *edem1* in the fat body (Figs S2A’ and B and 2). Hence, Edem1 function in the fat body acts in an IPC independent manner to maintain metabolic status. Here too, the exact molecular mechanism by which Edem1 regulates *upd2* transcript levels is not understood at the moment. Although our experiments rule out the role of Upd2 in regulating insulin signalling in response to *edem1*-RNAi, we do not know how enhancement of *upd2* levels in response to down-regulation of Edem1 in the fat body leads to IPC independent regulation of metabolic status.

Edem1 function in the fat body maintains systemic insulin signalling, and reduction in *edem1* levels in the fat body resulted in low systemic insulin signalling in larvae, which led to metabolic phenotypes as seen on circulating sugar levels and enhanced feeding in larvae; lipid and glycogen stores enhanced resistance to starvation and increase in life span in adult flies (Fig 1). Low insulin signalling has been reported to cause these phenotypes by previous studies (Böhni et al, 1999; Tatar et al, 2001; Rulifson et al, 2002; Broughton et al, 2005; Shingleton et al, 2005; Wu et al, 2005; Teleman et al, 2006; Slaidina et al, 2009; Grönke et al, 2010; Haselton et al, 2010; Varghese et al, 2010; Bai et al, 2012; Hong et al, 2012). We also show that the impact of reducing *edem1* levels on insulin signalling is due to the accumulation of DILP2 protein in the IPCs (Fig 2C) and reduced *dilp3* transcript levels in the larval IPCs (Fig 2B). However, it should be noted that we did not observe any changes at the protein and mRNA levels of other mNSC DILPs. DILPs are known to be regulated in a context specific manner, gene expression as well as protein levels in IPCs vary based on nutritional cues, developmental stages and various neural and endocrine signals that act on the IPCs (Ikeya et al, 2002; Grönke et al, 2010; Varghese et al, 2010; Hong et al, 2012; Söderberg et al, 2012; Luo et al, 2014; Kim & Neufeld, 2015; Hallier et al, 2016). Eiger activity on IPCs in response to low protein diet has been shown to suppress *dilp2* and *dilp5* transcript levels (Agrawal et al, 2016). However, here we show that enhanced Eiger levels (Fig 8A and B) due to suppression of Edem1 expression in fed conditions affects *dilp3* transcription and DILP2 protein accumulation in the IPCs (Fig 8C–E). Strictly the roles of individual DILPs are not understood; however, the effects of ablating IPCs, on growth and metabolism could be rescued by DILP2 expression alone (Rulifson et al, 2002; Haselton et al, 2010). Many reports hint at effects on insulin signalling caused by an individual DILP or more than one DILP (Bai et al, 2012; Sudhakar et al, 2019).

Here, it may be noted that reduction in Edem1 levels in the fat body did not cause any effects on larval growth or developmental delays. One possible explanation for the lack of growth and developmental phenotypes is that the reduction in insulin signalling is not too drastic in response to lowering Edem1 levels in the fat body (Fig 2), in comparison to what is observed in response to insulin signalling pathway mutants or ablation of IPCs. However, the single and double mutants of both *dilp2* and *dilp3* produced very mild growth phenotypes (Grönke et al, 2010). Here, we report changes in the levels of *dilp3* transcripts and DILP2 accumulation in the IPCs, which leads to no effects on body size, unlike complete loss of IPC function. Also, minimal reduction in insulin signalling in an *inr* mutant background showed elevated lipid and glycogen levels, whereas showing no effects on body size (Shingleton et al, 2005). Another possibility is the fact that the expression of *edem1-RNAi* in the fat body enhanced feeding responses (Fig 1G), which may compensate for the growth effects caused by reduced insulin levels.

**Figure S4.**
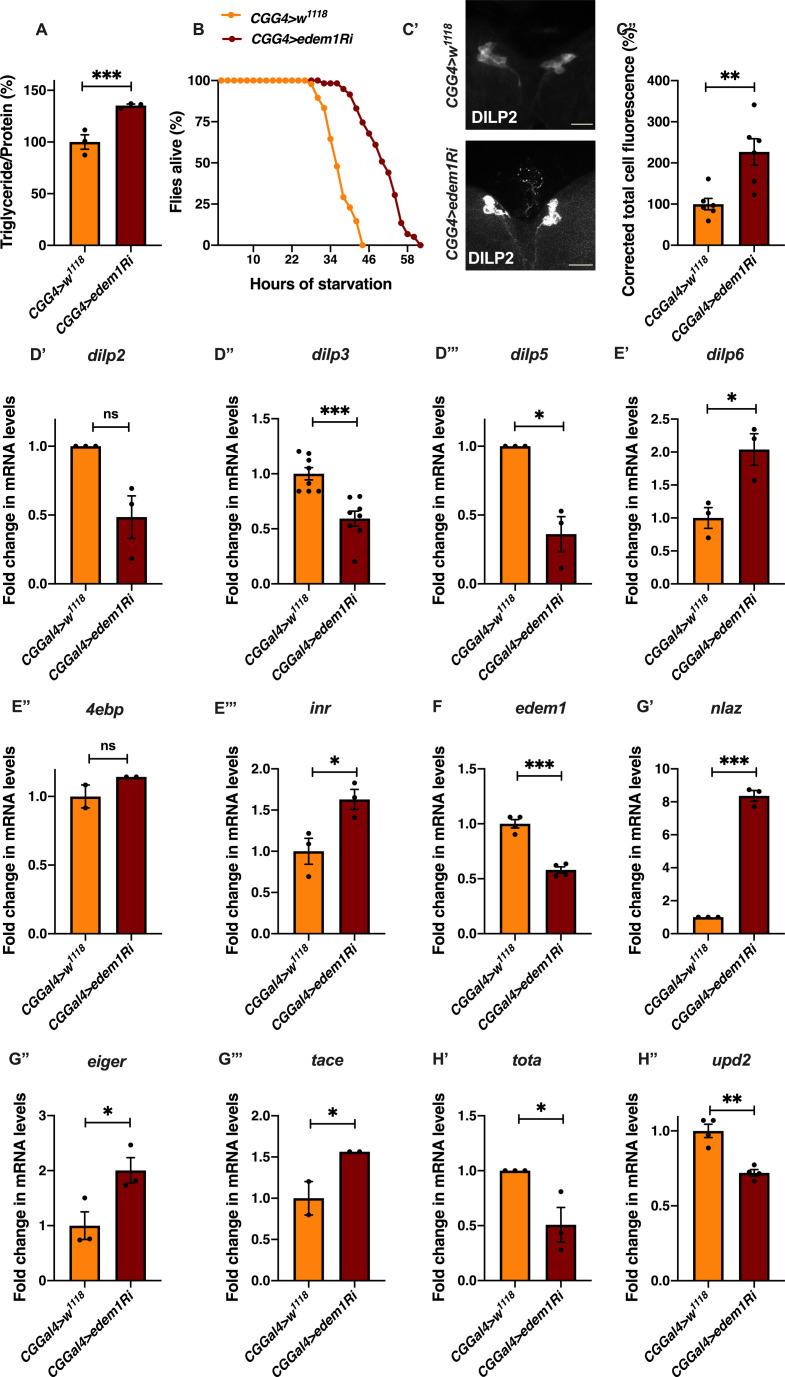
Blocking *edem1* in the fat body using *CgGal4* reduced insulin signalling and metabolic phenotypes. **(A)** Blocking *edem1* expression using RNAi in the fat body led to enhanced triglyceride levels in adult male flies. Data are shown as % ratio of triglyceride to total protein levels, normalised to 100% in *CgGal4>w*^*1118*^ (control) and increase in experimental conditions *CgGal4>UAS-edem1-RNAi* (independent biological replicates = 3, *P*-value between control and *UAS-edem1-RNAi* is <0.001 [Welch’s *t* test]). **(B)** Enhanced resistance to starvation in adult male flies caused by blocking *edem1* expression in the fat body. Data shown as percentage of flies of *CgGal4>w*^*1118*^ (control) and *CgGal4>UAS-edem1-RNAi* which were alive at various time points of starvation (independent biological replicates = 3. *P*-value between control and *UAS-edem1-RNAi* is <0.001 [log-rank test], Wald test = 65.66 on df = 1, *P* < 0.001 [Cox hazard proportional analysis]). **(C)** DILP2 protein in the larval insulin-producing cells shown as a representative image (C’) of anti-DILP2 antibody staining in larval brains of *CgGal4>w*^*1118*^ and *CgGal4>UAS-edem1-RNAi* (independent biological replicates = 6). Corrected total cell fluorescence values are normalised to *CgGal4>w*^*1118*^ and fold change in *CgGal4>UAS-edem1-RNAi* is shown in (C’’) (*P*-value between control and *UAS-edem1-RNAi* is 0.0089 [Welch’s *t* test]) (Scales: 25 µm). **(D)** Blocking *edem1* expression using RNAi in the fat body also led to a decrease in the levels of insulin-producing cell specific *dilp3* mRNA in larvae. Data are shown as fold change in mRNA levels, values are normalised to *CgGal4>w*^*1118*^, and fold change in *CgGal4>UAS-edem1-RNAi* is shown (n = 3, *P*-value between control and *UAS-edem1-RNAi* is 0.1 for *dilp2* [Mann–Whitney test], 0.0004 for *dilp3* [Welch’s *t* test] and 0.1 for *dilp5* [Mann–Whitney test]). **(E)** Blocking *edem1* expression using RNAi in the fat body led to an increase in mRNA levels of insulin target genes *dilp6* (E’), *4ebp* (E’’) and *inr* (E’’’) in larvae. Data are shown as fold change in mRNA levels, values are normalised to *CgGal4>w*^*1118*^, and fold change in *CgGal4>UAS-edem1-RNAi* is shown (independent biological replicates = 3, *P*-value between control and *UAS-edem1-RNAi* is 0.0284 for *dilp6* [Welch’s *t* test], 0.3333 for *4ebp* independent biological replicates = 2 [Mann–Whitney test] 0.0374 for *inr* [Welch’s *t* test]). **(F)** Down-regulation of *edem1* in the fat body led to decreased levels of *edem1* mRNA when compared with control. Data are normalised to *CgGal4>w*^*1118*^ and fold change in *CgGal4>UAS-edem1-RNAi* is shown (independent biological replicates = 4. *P*-value between control and *UAS-edem1-RNAi* is 0.0002 [Mann–Whitney test]). **(G)** Blocking *edem1* expression using RNAi in the fat body led to an increase in mRNA levels of insulin target genes *nlaz* (G’), *eiger* (G’’), and *tace* (G’’’) in larvae. Data are shown as fold change in mRNA levels, values are normalised to *CgGal4>w*^*1118*^, and fold change in *CgGal4>UAS-edem1-RNAi* is shown (independent biological replicates = 3, *P*-value between control and *UAS-edem1-RNAi* is <0.0001 for *nlaz* [unpaired *t* test], 0.0429 for *eiger* [Welch’s *t* test], and 0.1080 for *tace*-independent biological replicates = 2 [Welch’s *t* test]). **(H)** Down-regulation of *edem1* in the larval fat body led to decrease in *totA* mRNA levels (H’). Data are shown as fold change in mRNA levels, values are normalised to *CgGal4>w*^*1118*^, and fold change in *CgGal4>UAS-edem1-RNAi* is shown (independent biological replicates = 3. *P*-value between control and *UAS-edem1-RNAi* is 0.0353 [unpaired *t* test]). Down-regulation of *edem1* in the larval fat body led to decrease in *upd2* mRNA levels (H’’). Data are shown as fold change in mRNA levels, values are normalised to *CgGal4>w*^*1118*^, and fold change in *CgGal4>UAS-edem1-RNAi* is shown (independent biological replicates = 4. *P*-value between control and *UAS-edem1-RNAi* is 0.0037 [Welch’s *t* test]). **(A, C’’, D, E, F, G, H’’)** (*P*-value *<0.05; ** <0.01, *** <0.001; Data information: In [A, C’’, D, E, F, G, H’’], data are presented as mean ± SEM). Source data are available for this figure.

As our genetic screen and characterization of Edem1 was performed using the *pplGal4* driver, which could be active in other tissues as well, we performed key experiments using an additional fat body driver *CgGal4*. We observed similar effects with both the Gal4 drivers, conforming that the effects we see with knocking down Edem1 is specific to fat body (Fig S4).

Managing insulin signalling during nutrient withdrawal is crucial for mobilisation of nutrient stores and survival. In response to starvation, we report that *edem1* transcripts are reduced (Fig 7A). Reduction in *edem1* transcripts during starvation enhances *eiger* and *nlaz* levels, which aids in lowering insulin signalling (Fig 7C–F). This helps the flies to survive acute nutrient deprivation by mobilising energy stores (Figs 7B and 8). Surprisingly, we did not see any decrease in the levels of cleaved form of Eiger by enhancing Edem1 levels in the fat body. The levels of s-Egr are very low in normal fed flies (Fig 3C) and even during starvation, we did not see any increase as expected in s-Egr levels, although *edem1* transcript levels are reduced. However, in response to starvation, we observe an increase in *eiger* transcript levels, which is attenuated by Edem1 overexpression. There is a concurrent change to *nlaz* levels during starvation, indicating an increase in JNK signalling, which is missing in Edem1 overexpressing flies. Hence, although there is significant Eiger processing in the context of reduced Edem1 levels in the fat body, in response to starvation, we are unable to document this and an increase in Eiger transcript levels seems to be the primary effect, which is rescued by Edem1 overexpression.

In *edem1*-RNAi flies, the triglyceride content of the flies is significantly higher as compared to controls, which makes them resist starvation as compared to their control counterparts. Whereas, flies with Edem1 overexpression when starved are unable to use triglyceride stores and hence died faster (Fig 7B). Thus, reduction of Edem1 levels plays an important role in eliciting survival responses to starvation and enhancing Edem1 levels affected this, probably because of a failure in reducing insulin signalling and triglyceride mobilisation (Fig 7B–D). Moreover, reducing Edem1 levels in fed conditions led to enhanced feeding responses, similar to starvation conditions, further suggesting an active role for Edem1 in survival against food deprivation (Fig 1G). However, it is not yet clear if the function of Edem1 in regulating Eiger activity in the IPCs and systemic insulin signalling has any links to the ER stress pathway. Reduced *edem1* levels during starvation could be an outcome of reduced ER stress in response to low protein synthesis. Furthermore, reduction in *edem1* could cause aggregation of misfolded proteins in the ER, which might be responsible for the changes we report here on Eiger levels. However, blocking few other essential components of ERAD mechanism did not give us any expected results (Fig S3B). Moreover, we did not observe any evidence for enhanced activity in the ER stress pathway in response to Edem1 down-regulation in the fat body. Hence, we are currently not sure whether Edem1 activity on managing systemic insulin signalling and nutrient homeostasis is linked to its ERAD functions. We also cannot completely rule out a direct effect of Edem1 in the fat body on metabolic pathways, which is responsible for the nutrient homeostasis defects we report here.

To summarize, we show that Edem1, a key ERAD regulator, aids in the maintenance of nutrient homeostasis by managing the activity of TNFα Eiger on *Drosophila* insulin-producing cells (Fig 8). During fed conditions, Edem1 suppresses Eiger levels, which allows optimal insulin signalling and maintain a steady metabolic status. In response to starvation, our data suggest that lower levels of Edem1 leads to a reduction in insulin signalling and mobilisation of energy reserves, which aids in survival during acute food deprivation.

## Materials and Methods

### Fly strains

Fly stocks were reared in vials with standard food which consisted of 5.8% cornmeal, 5% dextrose, 2.36% yeast, 0.8% agar, and 10% Nipagen in 100% ethanol. All the flies were maintained at 25°C with 12:12 h light:dark cycle. *Cg*Gal4 (RRID:BDSC_7011), *UAS-InR*^*A1325D*^ (RRID:BDSC_8263), and *UAS-edem1-RNAi* (RRID:BDSC_58298) were obtained from Bloomington Drosophila stock center (BDSC). The RNAi lines used were obtained from Vienna Drosophila resource center (VDRC): *UAS-edem1-RNAi* (stock #6923, 6922), *UAS-eiger-RNAi* (stock #45253), *UAS-grnd-RNAi* (stock #43454), *UAS-dilp6-RNAi* (GD) (stock #45218), *UAS-herp-RNAi* (stock #11724, 11725), and *UAS-sip3-RNAi* (stock #6870, 107060). *dilp2*-Gal4/*CyO*GFP, *pumpless*-Gal4, and *w*^*1118*^ were obtained from Stephen Cohen. *UAS-dEDEM1* was from Koichi Iijima, *UAS-rheb* was obtained from Jagat. K. Roy, and *UAS-upd2-EGFP/TM3Sb* and 10XSTAT92E-GFP were obtained from Akhila Rajan. To match the genetic background all the fly strains used in this study were back-crossed into an isogenic *w*^*1118*^ background for at least six generations.

### Triglyceride and glycogen measurements

All experiments were carried out in controlled growth conditions as described here, unless mentioned otherwise. Fifty 1^st^ instar larvae were collected in fresh food vials avoiding overcrowding within 2–3 h of hatching. GFP balancers were used wherever required to aid in genotyping. Freshly emerged adult male flies were collected (15 per vial) and 5-d-old flies were used for triglyceride and glycogen measurement unless mentioned otherwise. Five flies in triplicates per genotype were homogenized in 0.05% Tween-20 using Bullet Blender Storm BBY24M from Next Advance. Each experiment was replicated independently and number of independent biological replicates is mentioned for each experiment in the figure legends. The homogenate was heat-inactivated at 70°C for 5 min and then centrifuged at 14,000 rpm for 3 min using Eppendorf 5424 centrifuge (FA-45-24-11 rotor). Serum triglyceride determination kit (Cat. no. TR0100) from Sigma-Aldrich was used to quantify triglyceride levels and protein levels were measured using the Quick Start Bradford 1× Dye Reagent (Cat. no. 500-0205) from Bio-Rad. This was followed by colorimetric estimation using TECAN Infinite M200 pro-multimode plate reader in 96-well format. The absorption maximum of 540 and 595 nm were used for triglyceride and protein content, respectively. Sample preparation for glycogen measurement was similar to triglycerides, following the manufacturer’s protocol (Cat. no. MAK016 from Sigma-Aldrich). The absorbance was measured at 570 nm. For triglyceride and glycogen utilisation assay, 5-d-old males (15 per vial) were transferred to vials containing 1% agar, were collected at the indicated time points, and homogenized as mentioned above. Each experiment was replicated independently and number of replicates (n) is mentioned for each experiment in the figure legends.

### Starvation sensitivity assay

Fifty 1^st^ instar larvae were collected in fresh food vials avoiding overcrowding within 2–3 h of hatching. GFP balancers were used wherever required to aid in genotyping. Freshly emerged adult male flies were collected (15 per vial). For starvation sensitivity assay, 15 (5 d old) male flies were transferred to vials containing 1% agar and the number of dead flies was counted every 2 h. Multiple vials were set as technical replicates. These experiments were replicated independently and number of independent biological replicates is mentioned in the figure legends.

### Life span assay

Adult life span assay was estimated with data obtained from three independent biological replicates for each genotype. Fifty 1^st^ instar larvae were collected in fresh food vials and freshly emerged adult male flies were collected (15 per vial). Multiple vials of adult male flies were set as technical replicates. These flies were flipped into fresh media every 2 d and the dead flies and the escapers were scored.

### Glucose assay

Fifty 1^st^ instar larvae were collected in fresh food vials. Larvae at third instar stage (five larvae for every prep) were used to isolate hemolymph using Zymo-Spin IIIC (C1006-250) from Zymo Research. 1 μl of hemolymph was diluted to 50 μl with autoclaved milli-Q water. 100 μl of glucose assay reagent (Cat. #no. AGO20) from Sigma-Aldrich was added and the reaction was incubated at 37°C for 30 min. The reaction was stopped with 100 μl of 12 N H_2_SO_4._ The glucose content was analyzed using colorimetric quantification at 540 nm using TECAN Infinite M200 pro-multi-mode plate reader in 96-well format. Hemolymph glucose measurements were replicated independently and number of replicates is mentioned in the figure legends.

### Larval starvation

Fifty 1^st^ instar larvae were collected in fresh food vials. Third instar non-crawling larvae of the desired genotypes were kept for starvation on 1% agar vials for 12 h, after washing them with milli-Q water to make sure that there were no traces of media left behind. After 12 h, the larvae were plunged for the qPCR experiments. The starvation experiments were replicated independently and number of replicates is mentioned in the figure legends.

### Feeding assay

Fifty 1^st^ instar larvae were collected in fresh food vials. Larvae at third instar stage (10 each) or 5-d-old flies (five each) were fed for 3 h or 30 min, respectively, with colored food with Orange G dye (Cat. no. 1936-15-8) from Sigma-Aldrich. The larvae/flies were homogenized using 0.05% Tween-20. The homogenate was analyzed colorimetrically at 492 nm using TECAN Infinite M200 pro-multi-mode plate reader in the 96-well format. The absorbance of the homogenate was directly proportional to the food intake. The feeding experiments were replicated independently and number of replicates is mentioned in the figure legends.

### Quantitative RT-PCR

Fifty 1^st^ instar larvae were collected in fresh food vials. Third instar wandering larvae or 5-d-old male flies for each genotype were collected and were flash-frozen. These experiments were replicated independently and number of replicates (n) is mentioned in the figure legends. Total RNA was isolated with QIAGEN RNeasy Plus Mini Kit (Cat. no. 74134) and was quantified using the Qubit RNA HS Assay Kit (Cat. no. Q32852). An equal amount of RNA from each sample was reverse-transcribed using SuperScript III First-Strand Synthesis System (Cat. no. 18080051) from Thermo Fisher Scientific. Quantitative RT-PCR was performed using Bio-Rad CFX96 with the cDNA template, Power SYBR Green PCR Master Mix (Cat. no. 4368702) from Thermo Fisher Scientific and a primer concentration of 312.5 nM. The data were normalised to *rp49*. The sequences of the primers used are mentioned in Table 1.

**Table 1.** List of primer sequences used in the manuscript.

*4ebp*	FP: 5′-CACTCCTGGAGGCACCA-3′ RP: 5′-GAGTTCCCCTCAGCAAGCAA-3′
*dilp2*	FP: 5′-GGCCAGCTCCACAGTGAAGT-3′ RP: 5′-TCGCTGTCGGCACCGGGCAT-3′
*dilp3*	FP: 5′-AAGCTCTGTGTGTATGGCTT-3′ RP: 5′-AGCACAATATCTCAGCACCT-3′
*dilp5*	FP: 5′-TCCGCCCAGGCCGCAAACTC-3′ RP: 5′-TAATCGAATAGGCCCAAGGT-3′
*dilp6*	FP: 5′-CGATGTATTTCCCAACAGTTTCG-3′ RP: 5′-AAATCGGTTACGTTCTGCAAGTC-3′
*edem1*	FP: 5′-CAATCCGGCACAAGCACTACCATGG-3′ RP: 5′-CCTCGCGTATCTGCTCGAAGTTGCT-3′
*eiger*	FP: 5′-CAGCTGATCCCCCTGGTTTT-3′ RP: 5′-GCCAGATCGTTAGTGCGAGA-3′
*inr*	FP: 5′-CACCCCGCTTCTATACTCCA-3′ RP: 5′-GTTAGGATGGTGGCCTGTTC-3′
*nlaz*	FP: 5′-GGTGAATGCGGCCATCAATC-3′ RP: 5′-AATGGCTGCGTCGGGTAAAA-3′
*rp49*	FP: 5′-GCTAAGCTGTCGCACAAA-3′ RP: 5′-TCCGGTGGGCAGCATGTG-3′
*tace*	FP: 5′-TGGGACACAATTTTGGAGCA-3′ RP: 5′-CCTCCTT GGTCCTCATTTGG-3′
*totA*	FP: 5′-AATTCTTCAACTGCTCTTATGTGC-3′ RP: 5′-TTTGGAGTCATCGTCCTGGG-3′
*upd2*	FP: 5′-CGGAACATCACGATGAGCGAAT-3′ RP: 5′-TCGGCAGGAACTTGTACTCG-3′

### Immunohistochemistry

DILP2 peptide corresponding to the sequence TRQRQGIVERC (amino acids 108–118) was used as an immunogen to raise DILP2 polyclonal antibody in rabbit (Eurogentec). Mouse anti-GFP (Cat. #632375 from Living Colors) was used. About 10 larvae (third instar wandering) were used to dissect the brains in ice-cold 1× phosphate-buffered saline PBS (Cat. no. P4417 from Sigma-Aldrich) per genotype for each experiment. The dissections were repeated independently and number of replicates (n) is mentioned in the figure legends. The tissue samples were fixed using 4% PFA (Cat #P6148 from Sigma-Aldrich) at room temperature for 20 min. PFA was removed and the tissues were washed with PBT 1× phosphate-buffered saline + 0.1% Triton X-100 (Cat. no. 161-0407 from Bio-Rad). Blocking solution (PBT + 0.1% BSA [Cat. no. A2153 from Sigma-Aldrich]) was added to the tissues and the tissues were incubated at room temperature for 45 min. Primary antibody against DILP2 and GFP were diluted in blocking solution in 1:1,000 and 1:500 dilutions, respectively. The samples were incubated with primary antibody overnight at 4°C with constant rotation. Then the tissues were washed extensively with PBT and incubated with secondary antibody at room temperature for 2 h. The secondary antibodies Alexa Fluor 488 Goat Anti-Rabbit Immunoglobulin G (IgG). (Cat. no. A27034) and Alexa Fluor 633 Goat Anti-Mouse IgG (Cat. no. A-21050) were diluted in 1:500 dilution in blocking solution. After 2 h the samples were washed extensively and mounted with a drop of SlowFade Gold Antifade Reagent with DAPI (Cat. no. S36939) from Thermo Fisher Scientific. The tissues were imaged using a Leica DM6000B upright microscope and processed using ImageJ software. Corrected total cell fluorescence was calculated using the formula Corrected total cell fluorescence = Integrated Density − (Area of selected cell × Mean fluorescence of background readings).

### Ex vivo organ co-culture

For the ex vivo organ co-culture, larval hemolymph was isolated from 10 (third instar crawling) larvae and was incubated with 10 brains from third instar crawling larvae of the desired genotypes in Shields and Sang medium (Cat. no. S3652 from Sigma-Aldrich) at room temperature for 2 h with constant shaking. The larval brains were then fixed in 4% PFA and stained for DILP2 as mentioned above and imaged.

### Western blotting

5-d-old adult flies five each of the desired genotype were homogenized in 40 μl RIPA buffer with cOmplete, EDTA-free Protease Inhibitor Cocktail (Cat. no. 4693132001 from Sigma-Aldrich). The homogenates were centrifuged at full speed. The samples were denatured in 2× Laemmli sample buffer (Cat. no. 1610737 from Bio-Rad) at 95°C and run on 10% SDS–PAGE. Immobilon Western Chemiluminescent HRP Substrate (Cat. no.WBKLS0050 from Merck; Millipore) was used for antibody detection after blotting on PVDF membrane (Cat. no. 162-0177 from Bio-Rad). The following primary antibodies were used: anti-Egr 1:50 (generous gift from Konrad Basler) and anti-actin 1:3,000 (Cat. no. 612656 from BD Biosciences). For quantification, the intensity of soluble Eiger protein bands was normalised to the intensity of actin bands using ImageJ software.

### Statistical analysis

All the experiments were carried out in biological replicates as indicated and the error bars represent the SEM. The graphs were plotted using GraphPad Prism8 software. Significance was tested using unpaired *t* test, Welch test, Mann–Whitney test and Kruskal–Wallis test (followed by Dunn’s post hoc test) with * representing *P*-value < 0.05, **P-value < 0.01, ***P-value < 0.001. The starvation and life span datasets were subjected to log-rank test followed by Cox proportional hazard analysis using R software to analyze the trends in the survival of flies in both the assays.

## Data Availability

Included as data file sets in the supplementary data section.

## Supplementary Material

Reviewer comments
