# Supplementary material for: Edem1 activity in the fat body regulates insulin signalling and metabolic homeostasis in Drosophila
Source: Life Sci Alliance. 2021 Jun 17;4(8):e202101079. doi: 10.26508/lsa.202101079 (PMC8321676; doi:10.26508/lsa.202101079)
Supplement: Supplementary file 1 [file LSA-2021-01079_SdataF1.pdf]

Table 1-1

Raw triglyceride/protein ratio of 5-day old adult control and edem1Ri males		
	pplG4>w^{1118}	*pplG4>edem1Ri*
**Set 1**	71.563208426782	112.82782587799
**Set 2**	84.496345203656	106.17428076571
**Set 3**	101.02668885736	134.41571231672
**Set 4**	103.23253561506	124.72883713440
**Set 5**	103.00478443069	119.07260623753
**Set 6**	93.276382783242	84.387269133137
**Set 7**	94.710255198517	101.47040477219
**Set 8**	204.23933859164	204.71797960317
**Set 9**	196.46413095818	246.632573477
**Set 10**	99.415972360105	104.85982357604
**Set 11**	101.79377221137	129.81292048883
**Set 12**	64.843346048027	112.26731145632
**Set 13**	82.956007573542	114.69533130257
**Set 14**	67.661954634525	112.23591736842
**Set 15**	70.826682286257	108.76547192577
**Set 16**	71.428310139303	132.70398276400
**Set 17**	89.060284681715	118.06044033405

Table 1-1

Raw glycogen values of 5-day old adult control and edem1Ri males		
	pplG4>w^{1118}	*pplG4>edem1Ri*
**Set 1**	107.82698483801	122.79156638397
**Set 2**	95.937317308349	129.64748578326
**Set 3**	101.95048249576	129.96636575532
**Set 4**	103.95942631974	149.41348862281
**Set 5**	92.447859328378	126.89145173903
**Set 6**	97.877929709743	113.64426661402

Table 1-1

**Time in hours**	*pplG4>w1118*	*pplG4>edem1Ri*
0	100	100
2	100	100
4	100	100
6	100	100
8	100	100
10	99.622641509434	99.618320610687
12	99.622641509434	99.618320610687
14	99.622641509434	99.618320610687
16	99.622641509434	99.618320610687
18	99.245283018867	99.618320610687
20	99.245283018867	99.618320610687
22	98.490566037735	99.618320610687
24	93.584905660377	99.618320610687
26	89.056603773584	99.618320610687
28	82.641509433962	99.236641221374
30	73.207547169811	97.709923664122
32	59.622641509434	94.656488549618
34	35.471698113207	86.259541984732
36	20.377358490566	76.717557251908
38	10.943396226415	56.870229007633
40	3.3962264150943	38.931297709923
42	1.1320754716981	23.282442748091
44	0	10.305343511450
46		4.5801526717557
48		3.0534351145038
50		1.1450381679389
52		0

Table 1-1

	pplG4>w^{1118}	*pplG4>edem1Ri*
0	100	140.75354932653
12	70.704404805242	90.853658536585
24	16.244994539497	42.182380779031
36	7.0440480524208	18.465598835092

Table 1-1

	pplG4>w^{1118}	*pplG4>edem1Ri*
0	100	132.50723673008
12	56.378384235538	96.027925264314
24	31.601677992600	62.468073249640
36	14.586461509883	31.990216869707

Table 1-1

Raw glucose values of 5-day old adult control and edem1Ri males		
	pplG4>w^{1118}	*pplG4>edem1Ri*
**Set 1**	117.47100348150	158.94131450883
**Set 2**	93.789070764969	149.09307738175
**Set 3**	96.181884629440	152.94004500952
**Set 4**	92.558041124083	147.56198426590

Table 1-1

**Raw feeding values of 5-day old adult control and edem1Ri males**		
	pplG4>w^{1118}	*pplG4>edem1Ri*
**Set 1**	64.694433631763	116.89373297002
**Set 2**	113.85753211366	155.89723627870
**Set 3**	121.44803425457	184.50759050214

Table 1-1

Time in days	*pplG4>*w^{1118}	*pplG4>edem1Ri*
**0**	100	100
**1**	100	100
**2**	100	100
**3**	100	100
**4**	99.116997792494	100
**5**	97.571743929359	98.655913978494
**6**	97.571743929359	97.043010752688
**7**	97.571743929359	97.043010752688
**8**	97.130242825607	97.043010752688
**9**	96.909492273730	97.043010752688
**10**	96.026490066225	97.043010752688
**11**	95.143487858719	97.043010752688
**12**	94.701986754966	97.043010752688
**13**	94.039735099337	95.430107526881
**14**	94.039735099337	94.892473118279
**15**	94.039735099337	94.892473118279
**16**	93.818984547461	94.354838709677
**17**	93.156732891832	94.354838709677
**18**	92.715231788079	94.354838709677
**19**	92.715231788079	94.354838709677
**20**	92.494481236203	93.279569892473
**21**	92.494481236203	93.279569892473
**22**	92.273730684326	92.741935483871
**23**	92.052980132450	92.741935483871
**24**	91.832229580574	92.741935483871
**25**	91.169977924944	92.204301075268
**26**	90.286975717439	90.860215053763
**27**	89.624724061810	90.860215053763
**28**	88.300220750551	90.860215053763
**29**	88.079470198675	90.322580645161
**30**	87.858719646799	90.322580645161
**31**	87.417218543046	90.322580645161
**32**	86.534216335540	89.784946236559
**33**	86.534216335540	89.784946236559
**34**	85.651214128035	89.784946236559
**35**	84.988962472406	87.903225806451
**36**	84.988962472406	87.903225806451
**37**	84.105960264900	86.290322580645
**38**	84.105960264900	86.290322580645
**39**	81.898454746136	86.290322580645
**40**	81.015452538631	85.215053763440
**41**	80.573951434878	84.408602150537
**42**	79.249448123620	84.408602150537
**43**	78.587196467991	83.333333333333
**44**	76.158940397351	82.795698924731
**45**	74.39293598234	82.795698924731
**46**	70.419426048565	80.107526881720
**47**	68.432671081677	76.881720430107
**48**	64.238410596026	76.881720430107
**49**	60.927152317880	75.537634408602
**50**	54.083885209713	73.924731182795
**51**	45.916114790287	73.924731182795
**52**	39.955849889624	71.774193548387
**53**	30.905077262693	68.548387096774
**54**	23.620309050772	68.548387096774
**55**	18.763796909492	66.935483870967
**56**	16.777041942604	63.172043010752
**57**	14.569536423841	63.172043010752
**58**	10.154525386313	60.483870967741
**59**	7.2847682119205	58.333333333333
**60**	6.8432671081677	58.333333333333
**61**	4.4150110375275	55.645161290322
**62**	4.1942604856512	48.387096774193
**63**	3.7527593818984	48.387096774193
**64**	3.3112582781457	43.548387096774
**65**	3.0905077262693	41.935483870967

Time in days	*pplG4>w¹¹¹⁸*	*pplG4>edem1Ri*
**66**	2.6490066225165	41.935483870967
**67**	0	36.559139784946
**68**		32.526881720430
**69**		32.526881720430
**70**		27.150537634408
**71**		24.193548387096
**72**		24.193548387096
**73**		21.774193548387
**74**		15.322580645161
**75**		15.322580645161
**76**		9.6774193548387
**77**		3.2258064516129
**78**		3.2258064516129
**79**		2.6881720430107
**80**		1.3440860215053
**81**		1.3440860215053
**82**		0.8064516129032
**83**		0.2688172043010
**84**		0.2688172043010
**85**		0.2688172043010
**86**		0
