# Supplementary material for: Edem1 activity in the fat body regulates insulin signalling and metabolic homeostasis in Drosophila
Source: Life Sci Alliance. 2021 Jun 17;4(8):e202101079. doi: 10.26508/lsa.202101079 (PMC8321676; doi:10.26508/lsa.202101079)
Supplement: Supplementary file 2 [file LSA-2021-01079_SdataFS1.pdf]

Table 1-1

Raw mRNA values in control and edem1Ri larvae		
	pplG4>w¹¹¹⁸	*pplG4>edem1Ri*
**Set 1**	0.9333	0.62617
**Set 2**	0.9333	0.42608
**Set 3**	1.01036	0.68658
**Set 4**	0.9333	0.50885
**Set 5**	1.06413	0.78433
**Set 6**	1.19229	0.81905
**Set 7**	0.9333	0.73833

Raw triglyceride/protein ratio of 5-day old adult control and edem1Ri males

	pplG4>w^{1118}	***pplG4>edem1Ri (6922)***	***pplG4>edem1Ri (BL58298)***	
**Set 1**	111.95178004370	129.96894367886	127.12502817190	
**Set 2**	84.469868312632	140.32037941789	169.40212348686	
**Set 3**	103.57835164366	130.458709876	168.94880985209	

Table 1-1

Percentage values of flies surviving after starvation of 5-day old adult control and edem1Ri males			
**Time in hours**	***pplG4>w1118***	***pplG4>edem1Ri (6922)***	***pplG4>edem1Ri (BL58298)***
**0**	100	100	100
**2**	100	100	100
**4**	100	100	100
**6**	100	100	100
**8**	100	100	100
**10**	100	100	100
**12**	100	100	100
**14**	100	100	100
**16**	100	100	100
**18**	100	100	100
**20**	100	100	100
**22**	100	100	100
**24**	87.5	98.387096774193	100
**26**	83.333333333333	98.387096774193	100
**28**	83.333333333333	98.387096774193	100
**30**	83.333333333333	96.774193548387	100
**32**	75	96.774193548387	100
**34**	66.666666666666	95.161290322580	100
**36**	62.5	95.161290322580	100
**38**	50	95.161290322580	93.220338983050
**40**	33.333333333333	93.548387096774	91.525423728813
**42**	33.333333333333	90.322580645161	86.440677966101
**44**	29.166666666666	88.709677419354	81.355932203389
**46**	20.833333333333	80.645161290322	77.966101694915
**48**	16.666666666666	66.129032258064	66.101694915254
**50**	12.5	53.225806451612	59.322033898305
**52**	0	43.548387096774	42.372881355932
**54**		33.870967741935	32.203389830508
**56**		30.645161290322	23.728813559322
**58**		20.967741935483	16.949152542372
**60**		14.516129032258	10.169491525423
**62**		8.0645161290322	5.0847457627118
**64**		1.6129032258064	3.3898305084745
**66**		0	3.3898305084745
**68**			1.6949152542372
**70**			1.6949152542372
**72**			1.6949152542372
**74**			1.6949152542372
**76**			1.6949152542372
**78**			0

Raw mRNA values in control, edem1Ri and edem1Ri-inrca larvae

**Raw mRNA values in control and edem1Ri larvae**			
**4ebp**	*pplG4>w1118*	*pplG4>edem1Ri*	*pplG4>edem1Ri-inrca*
Set 1	0.90557	1.09245	0.98642
Set 2	1.02084	1.18816	1.09245
Set 3	0.98314	1.31815	1.09245
Set 4	1.09045	1.38236	1.09245
dilp3	1.02638	0.47757	0.61978
Set 1	1.0483	0.49682	1.09772
Set 2	0.95317	0.44931	1.14241
Set 3	0.97215	0.5211	1.40766
dilp6	0.9253	1.56794	1.15085
Set 1	0.83643	1.56794	1.14414
Set 2	1.26363	1.56794	0.97383
Set 3	0.97463	1.56794	1.12884

Raw CTCF values in control, edem1Ri and edem1Ri-inrca larval brains

**Raw CTCF values in control and edem1Ri larvae**			
	pplG4>w^{1118}	*pplG4>edem1Ri*	*pplG4>edem1Ri-inrca*
0	119.00035574253	1510.2600313615	95.270726792586
12	68.630447537754	795.57988373309	112.56633107879
24	93.247716419516	1384.1489632596	112.12304957834
36	119.12148030019	1273.8061722581	123.94425745376
