# Supplementary material for: Edem1 activity in the fat body regulates insulin signalling and metabolic homeostasis in Drosophila
Source: Life Sci Alliance. 2021 Jun 17;4(8):e202101079. doi: 10.26508/lsa.202101079 (PMC8321676; doi:10.26508/lsa.202101079)
Supplement: Supplementary file 3 [file LSA-2021-01079_SdataF2.pdf]

Table 1-1

Raw mRNA values in control and edem1Ri larvae		
dilp6	*pplG4>w*^{1118}	*pplG4>edem1Ri*
**Set 1**	0.91614	1.67526
**Set 2**	1.11284	1.60993
**Set 3**	0.97103	1.3619
4ebp		
**Set 1**	0.5779721367376	0.5973731508887
**Set 2**	0.9643310318363	2.1343223785476
**Set 3**	1.2992796104338	1.3116979801683
**Set 4**	1.2270605429993	1.5307489328452
**Set 5**	0.8043681912253	1.2992796104338
**Set 6**	0.8104217074975	1.7349571578376
**Set 7**	0.7047789380730	1.8742602404102
**Set 8**	1.0132286203292	1.5061530168058
**Set 9**	1.2992796104338	1.4921099343036
**Set 10**	1.2992796104338	0.4706591351009
inr		
**Set 1**	1.1001029349244	4.066689798704
**Set 2**	0.1697804290014	1.4240341613535
**Set 3**	0.3742911244805	1.4240341613535
**Set 4**	0.2356547048256	1.4240341613535
**Set 5**	1.1596622192982	1.7613166524701
**Set 6**	1.7613166524701	2.3130729286282
**Set 7**	1.2531500619911	1.7613166524701
**Set 8**	1.1847252205381	1.7613166524701
**Set 9**	1.7613166524701	3.1913744783262

Table 1-1

Raw mRNA values in control and edem1Ri larvae		
dilp2	*pplG4>w1118*	*pplG4>edem1Ri*
**Set 1**	1.11361	0.89628
**Set 2**	0.5959	0.94856
**Set 3**	0.96105	1.39527
**Set 4**	1.20869	1.18174
**Set 5**	0.51324	1.00569
**Set 6**	1.11361	1.48096
**Set 7**	1.26669	1.12539
**Set 8**	1.11361	1.29284
**Set 9**	1.11361	1.21915
dilp3		
**Set 1**	1.2931	1.12077
**Set 2**	0.92026	0.6755
**Set 3**	0.24283	0.39179
**Set 4**	1.51235	0.74273
**Set 5**	0.42832	0.21036
**Set 6**	0.99009	0.5879
**Set 7**	1.63289	0.79962
**Set 8**	0.99009	0.41396
**Set 9**	0.99009	0.50765
dilp5		
**Set 1**	1.09374	1.0448
**Set 2**	0.47766	1.09336
**Set 3**	0.4168	1.37038
**Set 4**	1.67067	1.22264
**Set 5**	0.29958	0.72826
**Set 6**	1.09374	0.97355
**Set 7**	1.76034	1.23507
**Set 8**	1.09374	1.189
**Set 9**	1.09374	0.55227

Table 1-1

Raw CTCF values in control and edem1Ri larvae		
	pplG4>w1118	*pplG4>edem1Ri*
**Set 1**	104.791402869072	336.803798140615
**Set 2**	116.272896785472	793.438703312624
**Set 3**	53.2067637188598	316.66830400633
**Set 4**	90.4773535356432	554.322045028149
**Set 5**	65.7725093819451	291.430899098655
**Set 6**	155.16184974317	321.001246721949
**Set 7**	153.622962219734	201.057201150194
**Set 8**	115.411238410223	186.644091425005
**Set 9**	145.629989841642	892.774479778175
**Set 10**	79.0039294329984	946.230038009585
**Set 11**	68.7660979125657	930.113567792912
**Set 12**	90.0484248212652	885.290443579294
**Set 13**	92.112201159701	580.615861139228
**Set 14**	82.3382028248983	396.858497855032
**Set 15**	87.3841773428094	

Table 1-1

Raw triglyceride/protein ratio of 5-day old adult control, edem1Ri, inrca, edem1Ri-inrca males				
	pplG4>w^{1118}	*pplG4>edem1Ri*	*pplG4>inrca*	*pplG4>edem1Ri-inrca*
**Set 1**	100.69686358597	157.17970781698	208.27543661761	118.081558657692
**Set 2**	82.225259840614	131.50762521783	165.28038701374	109.074720917442
**Set 3**	103.54010509595	138.88279160302	177.01635101532	94.5906672914431
**Set 4**	111.28147623121	124.30006632189	183.89315803621	96.3491439093725

Table 1-1

**Percentage values of flies surviving after starvation of 5-day old adult control, edem1Ri, inrca and edem1Ri-inrca males**				
	pplG4>w^{1118}	*pplG4>edem1Ri*	*pplG4>inrca*	*pplG4>edem1Ri-inrca*
**0**	100	100	100	100
**2**	100	100	100	100
**4**	100	100	100	100
**6**	100	100	100	100
**8**	100	100	100	100
**10**	100	100	100	100
**12**	100	100	100	100
**14**	100	100	100	100
**16**	100	100	100	100
**18**	100	100	100	100
**20**	100	100	100	100
**22**	100	100	100	100
**24**	97.029702970297	100	98.412698412698	99.21875
**26**	96.039603960396	100	96.825396825396	98.4375
**28**	96.039603960396	100	96.825396825396	96.09375
**30**	96.039603960396	99.090909090909	96.825396825396	94.53125
**32**	94.059405940594	99.090909090909	96.825396825396	92.96875
**34**	92.079207920792	97.272727272727	95.238095238095	85.15625
**36**	91.089108910891	95.454545454545	93.650793650793	71.875
**38**	87.128712871287	94.545454545454	92.063492063492	60.9375
**40**	79.207920792079	92.727272727272	92.063492063492	45.3125
**42**	76.237623762376	87.272727272727	88.888888888888	30.46875
**44**	62.376237623762	80	87.301587301587	20.3125
**46**	46.534653465346	78.181818181818	85.714285714285	10.15625
**48**	33.663366336633	69.090909090909	77.777777777777	6.25
**50**	29.702970297029	50.909090909090	68.253968253968	0
**52**	13.861386138613	41.818181818181	60.317460317460	
**54**	3.9603960396039	29.090909090909	55.555555555555	
**56**	0.9900990099009	22.727272727272	42.857142857142	
**58**	0	18.181818181818	39.682539682539	
**60**		13.636363636363	31.746031746031	
**62**		11.818181818181	22.222222222222	
**64**		10.909090909090	9.5238095238095	
**66**		10	6.3492063492063	
**68**		7.2727272727272	4.7619047619047	
**70**		2.7272727272727	4.7619047619047	
**72**		0	4.7619047619047	
**74**			4.7619047619047	
**76**			4.7619047619047	
**78**			3.1746031746031	
**80**			1.5873015873015	
**82**			1.5873015873015	
**84**			1.5873015873015	
**86**			1.5873015873015	
**88**			1.5873015873015	
**90**			1.5873015873015	
**92**			0	
