# Supplementary material for: Edem1 activity in the fat body regulates insulin signalling and metabolic homeostasis in Drosophila
Source: Life Sci Alliance. 2021 Jun 17;4(8):e202101079. doi: 10.26508/lsa.202101079 (PMC8321676; doi:10.26508/lsa.202101079)
Supplement: Supplementary file 4 [file LSA-2021-01079_SdataFS2.pdf]

Table 1-1

Raw mRNA values in control and edem1Ri larvae		
**tota**	*pplG4>w¹¹¹⁸*	*pplG4>edem1Ri*
**Set 1**	0.4988729778620	0.7641087740020
**Set 2**	1.2904496980252	0.2334426212607
**Set 3**	0.5510052040486	0.2536519655939
**Set 4**	0.7801092933119	1.1948700012871
**Set 5**	2.0179321122889	0.3650443427124
**Set 6**	0.8616307144630	0.3966465701844
upd2		
**Set 1**	0.9663391850539	0.3401513931389
**Set 2**	0.9663391850539	0.3872604284103
**Set 3**	1.0673216298921	0.4119793847640

Raw CTCF values in control and edem1Ri larvae

	pplG4>w¹¹¹⁸	*pplG4>edem1Ri*	
**Set 1**	43.968334869527	24.887131989468	
**Set 2**	173.74298361667	56.040871345709	
**Set 3**	91.910064942378	113.72123211883	
**Set 4**	105.17160496636	148.71017193344	
**Set 5**	36.995263173779	37.773326916789	
**Set 6**	61.320160521092	39.085502549589	
**Set 7**	84.143370529623	45.915058950905	
**Set 8**	131.39163614290	35.403877632645	
**Set 9**	15.922754858309	37.016927865130	
**Set 10**	95.906017277230	39.684834056676	
**Set 11**	76.551997156409	33.721312339698	
**Set 12**	77.628845240798	34.659639856480	
**Set 13**	188.60251411047	37.676885430629	
**Set 14**	97.044318694739	42.435181927338	
**Set 15**	110.70338336547	41.785923160979	
**Set 16**	131.33725710181	54.164930113215	
**Set 17**	177.65949343241		

Table 1-1

Raw triglyceride/protein ratio of 5-day old adult control, edem1Ri, upd2 and edem1Ri; upd2 males				
	ppIG4>w^{1118}	***ppIG4>edem1Ri***	***ppIG4>upd2***	***ppIG4>edemRi; upd2***
**Set 1**	100	149.95149595440	167.38608832513	112.31496218785
**Set 2**	79.685283027064	148.80742211147	185.25576713862	77.141484836070
**Set 3**	110.83253897727	116.22155800564	173.58211579902	70.964965960162
**Set 4**	110.43814016592	137.09579331770	186.09737213259	103.20838760218

Percentage values of flies surviving after starvation of 5-day old adult control, edem1Ri, upd2 and edem1Ri; upd2 males

Time in hours	***ppIG4>w*** w^{1118}	***ppIG4>edem1Ri***	***ppIG4>upd2***	***ppIG4>edemRi; upd2***		
**0**	100	100	100	100		
**2**	100	100	100	100		
**4**	100	100	100	100		
**6**	100	100	100	100		
**8**	100	100	100	100		
**10**	100	100	100	100		
**12**	100	100	100	100		
**14**	100	100	100	100		
**16**	100	100	100	100		
**18**	100	100	100	98.484848484848		
**20**	100	100	100	98.484848484848		
**22**	100	100	100	98.484848484848		
**24**	96.470588235294	100	97.560975609756	96.969696969697		
**26**	95.294117647058	100	95.121951219512	95.454545454545		
**28**	95.294117647058	100	95.121951219512	92.424242424242		
**30**	95.294117647058	100	95.121951219512	90.909090909090		
**32**	92.941176470588	100	95.121951219512	90.909090909090		
**34**	90.588235294117	100	90.243902439024	90.909090909090		
**36**	89.411764705882	98.989898989899	87.804878048780	90.909090909090		
**38**	84.705882352941	98.989898989899	85.365853658536	89.393939393939		
**40**	75.294117647058	97.979797979798	85.365853658536	74.242424242424		
**42**	71.764705882352	94.949494949494	82.926829268292	48.484848484848		
**44**	56.470588235294	87.878787878787	73.170731707317	34.848484848484		
**46**	43.529411764705	85.858585858585	63.414634146341	24.242424242424		
**48**	38.823529411764	75.757575757575	56.097560975609	18.181818181818		
**50**	34.117647058823	56.565656565656	48.780487804878	10.606060606060		
**52**	15.294117647058	45.454545454545	34.146341463414	4.5454545454545		
**54**	0	31.313131313131	19.512195121951	1.5151515151515		
**56**		24.242424242424	9.7560975609756	0		
**58**		20.202020202020	9.7560975609756			
**60**		15.151515151515	2.4390243902439			
**62**		13.131313131313	0			
**64**		12.121212121212				
**66**		11.1111111111111				
**68**		8.0808080808080				
**70**		3.0303030303030				
**72**		0				
**74**						

Raw mRNA values in control, edem1Ri, edem1Ri; upd2 larvae

4ebp	***pplG4>w^{1118}***	***pplG4>edem1Ri***	***pplG4>edemRi; upd2***
**Set 1**	1.32594	1.8376	1.28928
**Set 2**	1.62905	2.19207	1.8376
**Set 3**	0.78026	1.84663	1.72086
**Set 4**	0.26475	2.0711	0.29117
dilp3			
**Set 1**	0.941	0.44783	0.71742
**Set 2**	0.94708	0.44799	0.78859
**Set 3**	1.19807	0.78736	1.09422
**Set 4**	1.12526	0.59873	0.60666
**Set 5**	0.78859	0.1284	0.09768
inr			
**Set 1**	1.23503	2.96071	2.86211
**Set 2**	0.8544	2.74751	1.89048
**Set 3**	0.91057	2.55362	2.31225

Raw triglyceride/Protein ratio of 5-day old control, edem1Ri, dilp6Ri and edem1Ri; dilp6Ri males

	pplG4>w^{1118}	***pplG4>edem1Ri***	***pplG4>dilp6Ri***	***pplG4>edem1Ri; dilp6Ri***	
**Set 1**	103.10698122432	153.40627093632	168.17986158806	238.357890292193	
**Set 2**	90.211392390430	153.77759561012	161.48560653003	194.91960420949	
**Set 3**	93.870139001591	156.18181423565	167.58983607520	171.464190589528	

Percentage values of flies surviving after starvation of 5-day old adult control, edem1Ri, dilp6Ri and edem1Ri; dilp6Ri males

	pplG4>w^1118	***pplG4>edem1Ri***	***pplG4>dilp6Ri***	***pplG4>edem1Ri; dilp6Ri***	
**0**	100	100	100	100	
**2**	100	100	100	100	
**4**	100	100	100	100	
**6**	100	100	100	100	
**8**	100	100	100	100	
**10**	100	100	100	100	
**12**	100	100	100	100	
**14**	100	100	100	100	
**16**	100	100	100	100	
**18**	99.130434782608	100	100	98.8636363636364	
**20**	99.130434782608	100	100	96.5909090909091	
**22**	99.130434782608	100	100	95.4545454545455	
**24**	94.782608695652	100	100	95.4545454545455	
**26**	93.043478260869	100	100	95.4545454545455	
**28**	92.173913043478	100	100	94.3181818181818	
**30**	87.826086956521	100	100	94.3181818181818	
**32**	73.913043478260	98.550724637681	100	90.9090909090909	
**34**	55.652173913043	94.202898550724	95.555555555555	85.2272727272727	
**36**	34.782608695652	85.507246376811	95.555555555555	72.7272727272727	
**38**	23.478260869565	74.637681159420	93.333333333333	57.9545454545455	
**40**	11.304347826087	57.971014492753	91.111111111111	40.9090909090909	
**42**	6.9565217391304	44.927536231884	86.666666666666	26.1363636363636	
**44**	6.0869565217391	35.507246376811	77.777777777777	18.1818181818182	
**46**	4.3478260869565	34.057971014492	75.555555555555	14.7727272727273	
**48**	3.4782608695652	28.985507246376	68.888888888888	13.6363636363636	
**50**	2.6086956521739	23.913043478260	66.666666666666	12.5	
**52**	0	20.289855072463	64.444444444444	12.5	
**54**		19.565217391304	64.444444444444	12.5	
**56**		17.391304347826	64.444444444444	12.5	
**58**		14.492753623188	62.222222222222	11.3636363636364	
**60**		10.869565217391	62.222222222222	9.09090909090909	
**62**		9.4202898550724	53.333333333333	6.81818181818182	
**64**		8.6956521739130	40	5.68181818181818	
**66**		7.9710144927536	35.555555555555	4.54545454545455	
**68**		5.7971014492753	24.444444444444	1.13636363636364	
**70**		2.1739130434782	13.333333333333	0	
**72**		0	13.333333333333		
**74**			8.888888888888		
**76**			4.444444444444		
**78**			2.222222222222		
**80**			0		
