# Supplementary material for: Edem1 activity in the fat body regulates insulin signalling and metabolic homeostasis in Drosophila
Source: Life Sci Alliance. 2021 Jun 17;4(8):e202101079. doi: 10.26508/lsa.202101079 (PMC8321676; doi:10.26508/lsa.202101079)
Supplement: Supplementary file 5 [file LSA-2021-01079_SdataF3.pdf]

Table 1-1

**Raw mRNA values in control and edem1Ri larvae**		
tace	*pplG4>w¹¹¹⁸*	*pplG4>edem1Ri*
**Set 1**	1.0361430011118	3.9059184426541
**Set 2**	1.2232371183659	1.4583065954022
**Set 3**	1.5669789809979	1.9874422756938
**Set 4**	0.9636784342198	1.2232371183659
**Set 5**	0.6675449602346	0.8577094026558
**Set 6**	1.3984291384582	1.6128503729366
**Set 7**	0.6494532832540	2.5296543607806
**Set 8**	0.4945350833575	3.0619616336716

Table 1-1

Raw mRNA values in control and edem1Ri larvae		
eiger	*pplG4>w1118*	*pplG4>edem1Ri*
**Set 1**	0.8232805635597	1.1628420790686
**Set 2**	0.8543051902293	1.1628420790686
**Set 3**	0.8595379795851	1.6275370307060
**Set 4**	1.3000341875571	1.7385884492570
**Set 5**	1.1628420790686	1.4816236066244

Raw values of soluble eiger levels in 5-day old control and edem1Ri adult males

	pplG4>w^{1118}	*pplG4>edem1Ri*		
**Set 1**	2.1385734591758	4.4780203807862		
**Set 2**	0.7978570509480	3.6932235773900		
**Set 3**	0.3570832210764	4.7138587967419		
**Set 4**	0.5707104579450	3.9838231530376		
**Set 5**	1.1357758108546	2.4336774058282		

Raw mRNA values in control, edem1Ri and edem1Ri; eigerRi larvae

nlaz	*pplG4>w1118*	*pplG4>edem1Ri*	*pplG4>edem1Ri; eigerRi*
**Set 1**	2.04855	3.1318	0.426678
**Set 2**	1.47052	2.52906	0.583234
**Set 3**	1.03019	5.98289	0.324869
**Set 4**	1.13105	4.57618	0.529867
**Set 5**	1.12955	3.2841	0.231765
**Set 6**	0.35563	0.60824	0.348612
**Set 7**	0.72555	2.04044	0.331255
**Set 8**	0.94584	4.02147	0.528796
**Set 9**	0.46556	1.65807	0.43248
**Set 10**	0.61432	0.94657	0.217789
**Set 11**	1.27642	1.17428	0.214354
**Set 12**	0.84568	2.15382	0.7843
**Set 13**	1.65065	2.55669	1.47052
**Set 14**	0.31047	1.98946	0.96269

Table 1-1

Raw triglyceride/protein ratio of 5-day old adult control, edem1Ri, eigerRi, edem1Ri-eigerRi males				
	pplG4>w1118	*pplG4>edem1Ri*	***pplG4>eigerRi***	***pplG4>edem1Ri; eigerRi***
**Set 1**	97.115874354537	151.54796170780	196.43739765665	98.1512586473149
**Set 2**	101.62287588460	162.85797688297	176.09904969570	80.8109569324275
**Set 3**	101.26124976085	167.64044967839	202.38196574095	86.4229602080207

Percentage values of flies surviving after starvation of 5-day old adult control, edem1Ri, eigerRi and edem1Ri;eigerRi males

	pplG4>w¹¹¹⁸	*pplG4>edem1Ri*	***pplG4>eigerRi***	***pplG4>edem1Ri ; eigerRi***		
**0**	100	100	100	100		
**2**	100	100	100	100		
**4**	100	100	100	100		
**6**	100	100	100	100		
**8**	100	100	100	100		
**10**	100	100	100	100		
**12**	100	100	100	100		
**14**	100	100	100	100		
**16**	100	100	100	100		
**18**	99.507389162561	100	100	100		
**20**	99.507389162561	100	100	100		
**22**	99.507389162561	100	100	99.570815450643		
**24**	96.551724137931	100	100	98.283261802575		
**26**	95.073891625615	100	100	96.566523605150		
**28**	92.610837438423	100	100	93.991416309012		
**30**	87.192118226601	99.563318777292	100	86.695278969957		
**32**	73.891625615763	97.816593886462	100	78.969957081545		
**34**	46.305418719211	93.449781659388	98.275862068965	59.656652360515		
**36**	29.556650246305	85.589519650655	98.275862068965	40.343347639485		
**38**	19.704433497536	70.305676855895	98.275862068965	21.888412017167		
**40**	8.3743842364532	53.711790393013	98.275862068965	10.729613733905		
**42**	5.4187192118226	41.921397379912	96.551724137931	7.2961373390557		
**44**	3.4482758620689	30.567685589519	94.827586206896	4.2918454935622		
**46**	2.4630541871921	24.454148471615	93.103448275862	3.4334763948497		
**48**	1.9704433497536	20.087336244541	86.206896551724	2.5751072961373		
**50**	1.4778325123152	15.720524017467	79.310344827586	2.1459227467811		
**52**	0	12.227074235807	72.413793103448	1.7167381974248		
**54**		11.790393013100	63.793103448275	1.2875536480686		
**56**		10.480349344978	51.724137931034	0.8583690987124		
**58**		8.7336244541484	36.206896551724	0.4291845493562		
**60**		6.5502183406113	25.862068965517	0.4291845493562		
**62**		5.6768558951965	13.793103448275	0		
**64**		5.2401746724890	8.6206896551724			
**66**		4.8034934497816	6.8965517241379			
**68**		3.4934497816593	3.4482758620689			
**70**		1.3100436681222	1.7241379310344			
**72**		0	0			
