# Supplementary material for: Edem1 activity in the fat body regulates insulin signalling and metabolic homeostasis in Drosophila
Source: Life Sci Alliance. 2021 Jun 17;4(8):e202101079. doi: 10.26508/lsa.202101079 (PMC8321676; doi:10.26508/lsa.202101079)
Supplement: Supplementary file 6 [file LSA-2021-01079_SdataF4.pdf]

Table 1-1

**Raw CTCF values in control, edem1Ri, eigerRi and edem1Ri; eigerRi larvae**				
	pplG4>w^{1118}	*pplG4>edem1Ri*	*pplG4>eigerRi*	*pplG4>edem1Ri;eigerRi*
**Set 1**	103.08943263007	148.40128216389	127.98591126547	88.4110491795928
**Set 2**	98.749405131028	175.65759950660	166.07598362164	78.8732324300954
**Set 3**	98.161162238897	146.86867801738	155.12239202896	135.077903461729

Table 1-1

Raw mRNA values in control, edem1Ri and edem1Ri; eigerRi larvae			
dilp3	*pplG4>w*^{1118}	*pplG4>edem1Ri*	*pplG4>edem1Ri; eigerRi*
**Set 1**	1.1297	0.94673	0.84482
**Set 2**	1.13699	0.98954	0.97551
**Set 3**	0.60557	0.67639	0.94673
**Set 4**	1.71253	0.49358	1.87963
**Set 5**	0.94673	0.15415	0.87889
**Set 6**	1.05279	0.94673	0.99155
**Set 7**	0.93848	1.06183	1.34147
**Set 8**	0.74359	0.93955	0.94673
**Set 9**	1.1133869451685	0.4201472674367	1.16756804009046
**Set 10**	0.8238783163527	0.4464946354549	1.01761620320971
**Set 11**	1.0637690666981	0.5279887583079	0.909954590165455
**Set 12**	0.8934436991041	0.3065970223740	0.833279299623297
**Set 13**	0.8391395298795	0.0957518074507	0.747449174415839

Table 1-1

Raw mRNA values in control, edem1Ri and edem1Ri; eigerRi larvae			
dilp6	*pplG4>w1118*	*pplG4>edem1Ri*	*pplG4>edem1Ri; eigerRi*
**Set 1**	0.803	1.0219	0.90314
**Set 2**	0.73197	0.93415	0.98093
**Set 3**	1.75904	2.79462	0.17356
**Set 4**	0.77547	1.15175	0.15864
**Set 5**	0.79614	0.92068	1.09888
**Set 6**	1.39607	1.6599	1.2607
**Set 7**	0.65069	1.16485	1.0219
**Set 8**	2.60692	13.38763	1.22818
**Set 9**	0.102	4.68795	1.33396
**Set 10**	1.20434	4.68795	0.23602
**Set 11**	0.17437	4.68795	0.21573
inr			
**Set 1**	2.23307	2.37054	0.15713
**Set 2**	0.00366	0.30964	0.91441
**Set 3**	1.19123	1.67007	0.91441
**Set 4**	1.6519	6.10644	1.5863
**Set 5**	0.25493	4.87789	3.55729
**Set 6**	0.56203	10.28096	0.68146
**Set 7**	0.35385	4.99059	3.96576
**Set 8**	1.74934	7.24305	3.96576

Raw glucose values of third instar control, edem1Ri and edem1Ri; eigerRi larvae

	pplG4>w^{1118}	*pplG4>edem1Ri*	*pplG4>edem1Ri; eigerRi*	
**Set 1**	106.18763201981	148.86371913467	116.020831815864	
**Set 2**	96.731371549275	144.80479277441	98.523199067667	
**Set 3**	97.080996430912	143.75591812950	97.1246995411174	
