# Supplementary material for: Edem1 activity in the fat body regulates insulin signalling and metabolic homeostasis in Drosophila
Source: Life Sci Alliance. 2021 Jun 17;4(8):e202101079. doi: 10.26508/lsa.202101079 (PMC8321676; doi:10.26508/lsa.202101079)
Supplement: Supplementary file 7 [file LSA-2021-01079_SdataF5.pdf]

Raw mRNA values in control, edem1Ri and edem1Ri; rheb larvae

eiger	*pplG4>w1118*	*pplG4>edem1Ri*	*pplG4>edem1Ri; rheb*
**Set 1**	0.6685363217269	1.5350889895381	0.674533674537069
**Set 2**	1.1601949151571	1.7991308938828	0.663665592420206
**Set 3**	0.8723255080713	1.8325901115411	0.61360654712138
**Set 4**	1.2747374113275	1.9621605006669	0.662185604471392
**Set 5**	1.0242058437170	1.7000169418515	0.737696794574762
nlaz			
**Set 1**	0.84928	2.38839	0.95574
**Set 2**	1.10714	4.70725	1.28451133868618
**Set 3**	0.54495	1.94082	1.51494019350685
**Set 4**	0.71908	1.10799	1.72129
**Set 5**	1.49409	1.37453	0.46207
**Set 6**	0.9899	2.52111	1.72129
**Set 7**	1.93214	2.99269	1.01027569851267
**Set 8**	0.36342	2.32873	1.03681796295829

Raw mRNA values in control, edem1Ri and edem1Ri; rheb larvae

4ebp	*pplG4>w1118*	*pplG4>edem1Ri*	*pplG4>edem1Ri; rheb*
**Set 1**	0.92614	1.28353	1.33914255136512
**Set 2**	1.13786	1.53112	1.15699719039187
**Set 3**	2.77893	3.32745	1.19582389160701
**Set 4**	0.8385	0.84619	0.739889319519637
**Set 5**	1.27974	1.10745	0.62412
**Set 6**	0.545	1.28983	1.25592
**Set 7**	0.53498	0.81379	1.161
**Set 8**	1.08717	1.47197	1.20848
**Set 9**	0.18492	1.44662	0.94002
**Set 10**	1.16595	1.28353	1.17096
**Set 11**	0.97712	1.32472	0.682
**Set 12**	0.54369	1.28353	1.07425
inr			
**Set 1**	2.65438	2.81779	1.9129983382125
**Set 2**	0.00436	0.36806	0.0809763501118359
**Set 3**	0.71256	0.62127	0.60641
**Set 4**	0.49296	1.36781	1.08693
**Set 5**	0.52536	0.82118	1.18911
**Set 6**	1.41598	1.98517	1.03910591552906
**Set 7**	1.3423922372969	5.3084290875260	1.14241869510128
**Set 8**	0.9286737387322	2.5768196308809	2.04766907349916
**Set 9**	0.9897266459239	3.739846535353	2.24015366197306
**Set 10**	0.9591621497475	3.1582950405364	2.14342768349719
**Set 11**	0.9744443978357	3.4488316631524	2.1912526419525

Raw mRNA values in control, edem1Ri and edem1Ri; rheb larvae

dilp3	*pplG4>w^{1118}*	*pplG4>edem1Ri*	*pplG4>edem1Ri; rheb*
**Set 1**	1.06786	0.8949	1.06896
**Set 2**	1.07475	0.93537	0.56474
**Set 3**	0.57242	0.63936	0.98201
**Set 4**	1.35958	0.8935	1.15538
**Set 5**	1.27695	0.67944	0.80868
**Set 6**	0.86492	0.80346	0.98159
**Set 7**	1.61878	0.46656	0.89492
**Set 8**	0.8949	0.14571	0.6469
**Set 9**	0.99516	0.8949	0.31157
**Set 10**	0.88711	1.00371	0.67425
**Set 11**	0.70289	0.88811	0.61957
**Set 12**	1.02129	0.40338	0.72894
**Set 13**	0.85776	0.29435	0.5102
**Set 14**	0.80563	0.09193	0.61929

Raw triglyceride/protein ratio of 5-day old adult control, edem1Ri, rheb and edem1Ri; rheb males

	pplG4>w1118	*pplG4>edem1Ri*	*pplG4>rheb*	*ppl>edem1Ri; rheb*
**Set 1**	98.164443679515		111.535817689171	101.939270267911
**Set 2**	98.941286946308	137.73748852885	176.659527902722	108.616625832804
**Set 3**	102.89426937417	161.83163917326	170.423097536669	105.441442834329

Percentage values of flies surviving after starvation of 5-day old adult control, edem1Ri, rheb and edem1Ri; rheb males

	ppIG4>w^{1118}	***ppIG4>edem1Ri***	***ppIG4>rheb***	***ppIG4>edemRi; rheb***	
**0**	100	100	100	100	
**2**	100	100	100	100	
**4**	100	100	100	100	
**6**	100	100	100	100	
**8**	100	100	100	100	
**10**	100	100	100	100	
**12**	100	100	100	100	
**14**	100	100	100	100	
**16**	100	100	100	100	
**18**	100	100	100	100	
**20**	100	100	100	100	
**22**	100	100	100	100	
**24**	97.029702970297	100	100	97.6190476190476	
**26**	96.039603960396	100	100	96.031746031746	
**28**	96.039603960396	100	98.305084745762	96.031746031746	
**30**	96.039603960396	99.090909090909	98.305084745762	96.031746031746	
**32**	94.059405940594	99.090909090909	98.305084745762	92.8571428571429	
**34**	92.079207920792	97.272727272727	98.305084745762	90.4761904761905	
**36**	91.089108910891	95.454545454545	94.915254237288	86.5079365079365	
**38**	87.128712871287	94.545454545454	93.220338983050	84.9206349206349	
**40**	79.207920792079	92.727272727272	91.525423728813	80.952380952381	
**42**	76.237623762376	87.272727272727	88.135593220339	77.7777777777778	
**44**	62.376237623762	80	84.745762711864	61.9047619047619	
**46**	47.524752475247	78.181818181818	77.966101694915	50	
**48**	34.653465346534	69.090909090909	74.576271186440	34.1269841269841	
**50**	30.693069306930	50.909090909090	69.491525423728	23.8095238095238	
**52**	14.851485148514	41.818181818181	55.932203389830	15.8730158730159	
**54**	3.9603960396039	29.090909090909	54.237288135593	6.34920634920635	
**56**	0.9900990099009	22.727272727272	40.677966101694	0	
**58**	0	18.181818181818	30.508474576271		
**60**		13.636363636363	27.118644067796		
**62**		11.818181818181	20.338983050847		
**64**		10.909090909090	18.644067796610		
**66**		10	15.254237288135		
**68**		7.2727272727272	8.4745762711864		
**70**		2.7272727272727	6.7796610169491		
**72**		0	5.0847457627118		
**74**			1.6949152542372		
**76**			0		
