# Supplementary material for: Edem1 activity in the fat body regulates insulin signalling and metabolic homeostasis in Drosophila
Source: Life Sci Alliance. 2021 Jun 17;4(8):e202101079. doi: 10.26508/lsa.202101079 (PMC8321676; doi:10.26508/lsa.202101079)
Supplement: Supplementary file 8 [file LSA-2021-01079_SdataF6.pdf]

Raw CTCF values in CHCB, CHTB, THCB and THTB larval brains				
	CHCB	CHTB	THCB	THTB
Set 1	168.45447851687	156.72034783852	203.215659116136	113.328238673776
Set 2	204.58687217234	135.80547099081	262.610057049605	124.787179816063
Set 3	130.07107577538	268.48554934054	207.150373874681	121.423977398857
Set 4	137.02449091729	285.34787956324	184.012471991727	143.683872799892
Set 5	210.47530495792	170.45639408315	157.149054818743	152.982538715136
Set 6	46.891637510790	137.31749484780	654.853321056248	604.666636537291
Set 7	111.83858112358	84.462684487884	138.626789325576	126.023512538952
Set 8	204.51902350174	115.09475922177	142.84445108651	120.696890516222
Set 9	25.836646477955	70.345918140587	206.513161651545	87.6368558693074
Set 10	85.421363002010	99.671340052883	373.41956912252	104.084425646899
Set 11	66.770187444527	119.57625513239	377.905167652381	107.626608887657
Set 12	44.913078114367	32.321267052252	236.717137015571	142.630840680103
Set 13	92.774871162292	43.835785371988	251.287071296747	
Set 14	54.654991407461		160.704256102934	
Set 15	29.278477617558		218.326784162784	
Set 16	92.120312484278		296.833692229479	
Set 17	77.707688209619		648.625334544746	
Set 18	30.431897906786		924.488097639286	
Set 19	50.742811366553		1174.11766545016	
Set 20	91.919316950572		1009.70634217497	
Set 21	45.749411643830		138.669256570402	
Set 22	176.28554453427		217.759537656587	
Set 23	121.53193720197			
