# Supplementary material for: Edem1 activity in the fat body regulates insulin signalling and metabolic homeostasis in Drosophila
Source: Life Sci Alliance. 2021 Jun 17;4(8):e202101079. doi: 10.26508/lsa.202101079 (PMC8321676; doi:10.26508/lsa.202101079)
Supplement: Supplementary file 9 [file LSA-2021-01079_SdataF7.pdf]

Raw mRNA values in control fed and starved larvae		
edem1	*pplG4>w^1118*	*pplG4>edem1Ri*
**Set 1**	0.91887	0.37178
**Set 2**	0.91887	0.87462
**Set 3**	0.91887	0.48728
**Set 4**	1.05328	0.91887
**Set 5**	0.91887	0.80973
**Set 6**	0.99473	0.31397
**Set 7**	0.91887	0.7039
**Set 8**	1.04767	0.33663
**Set 9**	1.17385	0.82134
**Set 10**	1.46065	0.91887
**Set 11**	0.91887	0.55872
**Set 12**	0.91887	0.61649
**Set 13**	0.91887	0.7039
**Set 14**	0.91887	0.87462

Percentage values of flies surviving after starvation of 5-day old adult control and edem1 males

	ppIG4>w^{1118}	*ppIG4>edem1*			
0	100	100			
2	100	100			
4	100	100			
6	100	100			
8	100	100			
10	100	100			
12	100	100			
14	100	100			
16	100	100			
18	100	100			
20	100	100			
22	100	100			
24	100	100			
26	100	100			
28	100	100			
30	98.360655737704	100			
32	98.360655737704	96.153846153846			
34	98.360655737704	92.307692307692			
36	98.360655737704	80.769230769230			
38	95.081967213114	69.230769230769			
40	91.803278688524	38.461538461538			
42	80.327868852459	11.538461538461			
44	57.377049180327	5.7692307692307			
46	47.540983606557	3.8461538461538			
48	34.426229508196	0			
50	18.032786885245				
52	6.5573770491803				
54	4.9180327868852				
56	0				

Raw triglyceride/protein ratio of 5-day old adult control and edem1 fed and starved males

	pplG4>w1118 fed	pplG4>w1118 starved		
**Set 1**	100.742808522004	6.70175624566196		
**Set 2**	96.8370466225796	5.72235193032061		
**Set 3**	102.420144855417	11.2220736232722		
	**pplG4>edem1 fed**	**pplG4>edem1 starved**		
**Set 1**	103.072139453579	47.1091294341		
**Set 2**	91.8763399218815	48.6838786183422		
**Set 3**	105.051520624539	35.3034222944505		

Raw mRNA values in control and edem1 fed and starved larvae

dilp3	pplG4>w^{1118} fed	pplG4>w^{1118} starved		
**Set 1**	0.889305564918503	0.516856098292625		
**Set 2**	1.0295623537706	0.638900010758472		
**Set 3**	0.992445019999075	0.866710100428348		
**Set 4**	1.0295623537706	0.68104650643587		
**Set 5**	1.0295623537706	0.330026212501167		
**Set 6**	1.0295623537706	0.289502638256756		
**Set 7**	1	0.553840261112206		
	**pplG4>edem1 fed**	**pplG4>edem1 starved**		
**Set 1**	1.25846495188442	0.881551524446565		
**Set 2**	1.57480828070398	0.852989788767798		
**Set 3**	1.54902803936556	1.69148486974627		
**Set 4**	1.22171987147835	0.85220450936373		
**Set 5**	1.57480828070398	0.852992410098946		
**Set 6**	1.45694398244432	1.02058457004573		
**Set 7**	1.43929556776343	1.02530127874484		

Raw mRNA values in control and edem1 fed and starved larvae

4ebp	***pplG4>w^{1118} fed***	***pplG4>w^{1118} starved***		
**Set 1**	0.93663	2.3573		
**Set 2**	0.88439	2.0573		
**Set 3**	0.83115	2.14593		
**Set 4**	1.34783	2.1373		
	pplG4>edem1 fed	***pplG4>edem1 starved***		
**Set 1**	0.96621628097320	0.734543349543129		
**Set 2**	0.90436476065792	0.69773299265288		
**Set 3**	1.07867916680244	0.695156804376453		
**Set 4**	1.05073979156643	0.737357221856964		
dilp6	***pplG4>w^{1118} fed***	***pplG4>w^{1118} starved***		
**Set 1**	0.3951	0.91766		
**Set 2**	0.91788	1.41466		
**Set 3**	1.41466	1.74838		
**Set 4**	1.41466	1.6302		
**Set 5**	1.18932	1.37231		
**Set 6**	1.10111492316369	8.90446553607581		
**Set 7**	0.23804487399243	6.75606351084199		
**Set 8**	1.17715290959676	6.39497149154818		
**Set 9**	1.15607447242741	5.50652243816095		
**Set 10**	0.99599152275732	4.35334801916959		
	pplG4>edem1 fed	***pplG4>edem1 starved***		
**Set 1**	0.18618731194905	0.283379553122363		
**Set 2**	0.62725519283769	0.41365625317316		
**Set 3**	0.96289323656393	0.372438062896562		
**Set 4**	1.41093825022276	0.271670500076202		
**Set 5**	1.24837066536333	0.406071508676923		
**Set 6**	1.48796845694233	0.0393779512430292		
**Set 7**	1.57985686507994	0.464994789650992		
**Set 8**	1.24201963536557	0.067321700604698		
**Set 9**	0.68376683776229	0.0180943701230018		
**Set 10**	0.57074354791310	0.0309373032423125		
inr	***pplG4>w^{1118} fed***	***pplG4>w^{1118} starved***		
**Set 1**	0.58089	1.22066		
**Set 2**	0.47273	1.22066		
**Set 3**	1.22066	0.80416		
**Set 4**	0.87032	1.82882		
**Set 5**	0.70826	1.82882		
**Set 6**	0.78929	2.35832		
**Set 7**	1.75836	2.58683		
**Set 8**	1.70623	2.12981		
**Set 9**	0.99248	1.78336		
**Set 10**	1.00892	1.82882		
**Set 11**	0.89186	1.82882		

4ebp	***pplG4>w^{1118} fed***	***pplG4>w^{1118} starved***		
	pplG4>edem1 fed	***pplG4>edem1 starved***		
**Set 1**	0.759217141489107	0.213799506952488		
**Set 2**	1.53559712453655	0.609054409928243		
**Set 3**	0.766124335971116	0.911773935774415		
**Set 4**	1.14720897579408	0.320327210140473		
**Set 5**	1.15076771768177	0.912509948301186		
**Set 6**	1.01955528667284	1.3660392496878		
**Set 7**	0.995655099677125	1.74240046845984		
**Set 8**	1.04345547366855	1.28742340616892		
**Set 9**	0.848962141456755	1.25198157218746		
**Set 10**	0.733456703052100	1.27739422448544		
**Set 11**	0.700069233705815	1.58795915858401		

Raw mRNA values in control and edem1 fed and starved larvae

eiger	***pplG4>w^{1118} fed***	***pplG4>w^{1118} starved***		
**Set 1**	0.90214977860489	1.28069049605339		
**Set 2**	1.06000128344991	1.08623499967914		
**Set 3**	0.90671886029647[illegible]	1.54117949047038		
**Set 4**	1.13113007764872	1.28478470127703		
	pplG4>edem1 fed	***pplG4>edem1 starved***		
**Set 1**	1.17662219288734	0.90074332500432		
**Set 2**	1.09484072729559	0.866861534109659		
**Set 3**	1.05118413401851	1.13584146590605		
**Set 4**	0.67735294579856[illegible]	0.994876872063237		

Raw mRNA values in control and edem1 fed and starved larvae

nlaz	***pplG4>w^{1118} fed***	***pplG4>w^{1118} starved***		
**Set 1**	0.80145	1.13324		
**Set 2**	0.90327	1.33727		
**Set 3**	0.91833	1.43703		
**Set 4**	0.61861	1.68749		
**Set 5**	0.91394	1.99131		
**Set 6**	1.36748	2.13986		
**Set 7**	1.31799	2.94772		
**Set 8**	1.15893	2.49204		
	pplG4>edem1 fed	***pplG4>edem1 starved***		
**Set 1**	0.88736514507909[illegible]	0.602583177005843		
**Set 2**	0.95212425699520[illegible]	0.841918767205008		
**Set 3**	0.82259884569218[illegible]	0.673207265095486		
**Set 4**	0.69775247788055[illegible]	0.897298229725942		
**Set 5**	0.94745240097462[illegible]	1.25368896938856		
**Set 6**	0.95680330048659[illegible]	1.00246530248471		
**Set 7**	1.44598256319584	1.93803681422544		
**Set 8**	1.2899210096959	1.62921994379398		
