# Supplementary material for: Edem1 activity in the fat body regulates insulin signalling and metabolic homeostasis in Drosophila
Source: Life Sci Alliance. 2021 Jun 17;4(8):e202101079. doi: 10.26508/lsa.202101079 (PMC8321676; doi:10.26508/lsa.202101079)
Supplement: Supplementary file 10 [file LSA-2021-01079_SdataFS3.pdf]

Raw mRNA values in control and edem1 larvae

| | | |
|---|---|---|
| **edem1** | *pplG4>*$w^{1118}$ | *pplG4>edem1Ri* |
| **Set 1** | 0.7454 | 38.51835 |
| **Set 2** | 1.30991 | 91.34935 |
| **Set 3** | 1.69998 | 124.62975 |
| **Set 4** | 0.24471 | 8.37057 |

Raw triglyceride/Protein ratio of 5-day old control, 6870, 10760, 11724 and 11725 males

| | *pplG4>w1118* | *pplG4>6870* | *pplG4>107060* | *pplG4>11724* | *pplG4>11725* |
|---|---|---|---|---|---|
| **Set 1** | 102.20184093743 | 155.23720015358 | 170.50406553185 | 140.70341921754 | 130.08606002325 |
| **Set 2** | 110.32937178195 | 99.912185348351 | 137.03478995380 | 111.67852819928 | 115.81319600173 |
| **Set 3** | 87.468787280606 | 100.88023230196 | 92.234517184062 | 92.758639642887 | 97.345958767447 |
