# Supplementary material for: Edem1 activity in the fat body regulates insulin signalling and metabolic homeostasis in Drosophila
Source: Life Sci Alliance. 2021 Jun 17;4(8):e202101079. doi: 10.26508/lsa.202101079 (PMC8321676; doi:10.26508/lsa.202101079)
Supplement: Supplementary file 11 [file LSA-2021-01079_SdataFS4.pdf]

Table 1-1

**Raw triglyceride/protein ratio of 5-day old adult control and edem1Ri males**		
	CgG4>w^{1118}	*CgG4>edem1Ri*
**Set 1**	111.69253698167	137.17451477918
**Set 2**	100.84824152613	132.42452459349
**Set 3**	87.459221492190	136.33350901384

Table 1-1

Percentage values of flies surviving after starvation of 5-day old adult control and edem1Ri males		
**Time in hours**	*CgG4>w1118*	*CgG4>edem1Ri*
**0**	100	100
**2**	100	100
**4**	100	100
**6**	100	100
**8**	100	100
**10**	100	100
**12**	100	100
**14**	100	100
**16**	100	100
**18**	100	100
**20**	100	100
**22**	100	100
**24**	100	100
**26**	100	100
**28**	97.91666666666	100
**30**	89.58333333333	100
**32**	83.33333333333	98.305084745762
**34**	64.58333333333	98.305084745762
**36**	47.91666666666	98.305084745762
**38**	29.16666666666	94.915254237288
**40**	22.91666666666	91.525423728813
**42**	14.58333333333	83.050847457627
**44**	0	74.576271186440
**46**		67.796610169491
**48**		61.016949152542
**50**		50.847457627118
**52**		44.067796610169
**54**		30.508474576271
**56**		13.559322033898
**58**		6.7796610169491
**60**		5.0847457627118
**62**		0

Table 1-1

Raw CTCF values in control and edem1Ri larvae		
	CgG4>w^{1118}	*CgG4>edem1Ri*
**Set 1**	96.910015561120	262.06787561687
**Set 2**	107.15252833606	224.94601811933
**Set 3**	83.770192355811	122.44918890785
**Set 4**	91.933807493913	155.63606422460
**Set 5**	59.051903732632	341.04899239431
**Set 6**	161.18155252045	254.23705692947

Table 1-1

Raw mRNA values in control and edem1Ri larvae		
dilp2	*CgG4>w¹¹¹⁸*	*CgG4>edem1Ri*
**Set 1**	1	0.57912
**Set 2**	1	0.69369
**Set 3**	1	0.18268
dilp3		
**Set 1**	0.84215667525777	0.6175811230435
**Set 2**	0.84215667525777	0.4855899421710
**Set 3**	0.84215667525777	0.2020606802289
**Set 4**	1.1843104454373	0.6802072973221
**Set 5**	1.1234437458814	0.6466983216032
**Set 6**	1.2036712990101	0.7947550684963
**Set 7**	0.9112748749410	0.7866568698933
**Set 8**	1.0508296089567	0.5283186854430
dilp5		
**Set 1**	1	0.44294
**Set 2**	1	0.52872
**Set 3**	1	0.11213

Table 1-1

Raw mRNA values in control and edem1Ri larvae		
dilp6	*CgG4>w^{1118}*	*CgG4>edem1Ri*
**Set 1**	1.2275461352755	2.3440899282068
**Set 2**	0.6983509963582	1.5675518638242
**Set 3**	1.0741028683661	2.2038688162363
4ebp		
**Set 1**	0.91654	1.14226
**Set 2**	1.08346	1.14226
inr		
**Set 1**	1.2177774562845	1.8283956189752
**Set 2**	0.6927988313233	1.4089685169212
**Set 3**	1.0894237123921	1.6523413415596

Table 1-1

Raw mRNA values in control and edem1Ri larvae		
edem1	*CgG4>w^{1118}*	*CgG4>edem1Ri*
**Set 1**	1.06046	0.53873
**Set 2**	0.97614	0.5253
**Set 3**	1.06046	0.62226
**Set 4**	0.90294	0.6353

Table 1-1

Raw mRNA values in control and edem1Ri larvae		
nlaz	*CgG4>w1118*	*CgG4>edem1Ri*
**Set 1**	1	7.7193
**Set 2**	1	8.73834
**Set 3**	1	8.63265
**eiger**		
**Set 1**	1.50291746445576	2.4660986980852
**Set 2**	0.7388914552875	1.7322590776677
**Set 3**	0.7581910802547	1.8154836244629
**tace**		
**Set 1**	0.79758	1.56498
**Set 2**	1.20242	1.5649791325025

Table 1-1

Raw mRNA values in control and edem1Ri larvae		
tota	*CgG4>w[1118]*	*CgG4>edem1Ri*
**Set 1**	1	0.80991
**Set 2**	1	0.43407
**Set 3**	1	0.28026
**upd2**		
**Set 1**	0.88609	0.72531
**Set 2**	1.07061	0.77341
**Set 3**	0.9727	0.71501
**Set 4**	1.07061	0.66624
